# Engineering and evaluation of precision-glycosylated clickable albumin nanoplatform for targeting the tumor microenvironment

**DOI:** 10.7150/thno.123973

**Published:** 2026-01-01

**Authors:** Ji Yong Park, Jinyeong Choi, Jeongbin Park, Jin Sil Kim, Young Chan Ann, Hyewon Chung, Jisu Park, Jiyoon Kim, Seung Hyeok Seok, Hongyoon Choi, Hyung-Jun Im, Yun-Sang Lee

**Affiliations:** 1 Department of Nuclear Medicine, Seoul National University Hospital, 03080, Seoul, Republic of Korea.; 2 Cancer Research Institute, Seoul National University, 03080, Seoul, Republic of Korea.; 3 Department of Nuclear Medicine, Seoul National University College of Medicine, 03080, Seoul, Republic of Korea.; 4 Institute of Radiation Medicine, Medical Research Center, Seoul National University College of Medicine, 03080, Seoul, Republic of Korea.; 5 Portrai Inc. Dongsullagil, 78-18, Jongno-gu, 03136, Seoul, Republic of Korea.; 6 School of Dentistry, Seoul National University, Seoul, 03080, Republic of Korea.; 7 Macrophage Lab, Department of Microbiology and Immunology, Seoul National University College of Medicine, Seoul 110-799, South Korea.; 8 Institute of Endemic Diseases, Seoul National University Medical Research Center (SNUMRC), Seoul, Republic of Korea.; 9 Department of Biomedical Sciences and Seoul National University College of Medicine, Seoul, Republic of Korea.; 10 Department of Molecular Medicine and Biopharmaceutical Sciences, Graduate School of Convergence Science and Technology, Seoul National University, Seoul 08826, Republic of Korea.; 11 Department of Applied Bioengineering, Graduate School of Convergence Science and Technology, Seoul National University, Seoul 08826, Republic of Korea.; 12 Research Institute for Convergence Science, Seoul National University, Seoul, 08826, Republic of Korea.

**Keywords:** albumin, click chemistry, glycosylation, spatial transcriptomics, positron emission tomography, tumor microenvironment, theranostics

## Abstract

**Rationale:** Glycosylation of drug delivery vehicles enables selective tumor microenvironment (TME) targeting but is limited by the lack of precise glycan control and unbiased evaluation of in situ targeting. We developed a clickable albumin nanoplatform engineered by distinct glycosylation for selective *in vivo* cell targeting (CAN-DGIT) with a defined number of sugar moieties and integrated spatial transcriptomics (ST) to map nanoparticle-TME interactions.

**Methods**: Albumin was functionalized with azadibenzocyclooctyne (ADIBO) at a controlled degree of functionalization (DOF), confirmed by MALDI-TOF and UV-vis spectroscopy, followed by conjugation of azide-functionalized mannose, galactose, or glucose via click chemistry. Nanoparticles were labeled with ^64^Cu or fluorescent dyes for PET imaging and *ex vivo* analysis in healthy and 4T1 tumor-bearing mice. ST based algorithms, spatial gene-image integration (SPADE), cell-type deconvolution (CellDART), and image-based molecular signature analysis (IAMSAM), were used to define TME clusters, associated cell populations, and glycan receptor gene signatures. Clodronate-loaded glycosylated albumins were tested for tumor-associated macrophage (TAM) depletion.

**Results:** Glycosylation type of CAN-DGIT dictated pharmacokinetics and targeting. Mannosylated albumin (Man-Alb) showed rapid hepatic retention via mannose receptors on Kupffer cells and TAMs; galactosylated albumin (Gal-Alb) exhibited rapid hepatobiliary clearance with the highest tumor-to-liver ratio; glucosylated albumin at the C6 position (Glc(6)-Alb) progressively accumulated in tumors, correlating with glucose transporter 1 (GLUT1)-expressing cancer cells. ST confirmed Man-Alb enrichment in extracellular matrix (ECM)/TAM-rich clusters (mannose receptor C-type 1, Mrc1-high) and Gal-/Glc-Alb uptake in glycolytic/hypoxic tumor clusters (Slc2a1-high). Man-Alb-clodronate achieved potent CD206+ TAM depletion without altering drug release kinetics.

**Conclusions:** Precisely tuned glycosylation enables programmable biodistribution and cell-type targeting of albumin nanoparticles in the TME. Integrating PET with ST provides a robust framework for mechanistic mapping of nanomedicine uptake. The CAN-DGIT platform offers a versatile strategy for developing targeted theranostic agents with immunomodulatory potential.

## Introduction

Glycosylation of drug delivery carriers is attracting attention as a promising tumor microenvironment (TME)-targeting strategy 1-4. This approach leverages the fact that glycosylation-related binding proteins, such as galectins or sialic acid-binding immunoglobulin-type lectins (siglecs), and glycosylation-recognizing receptors tend to be overexpressed in the TME 5. Notably, glucose transporter subtype 1 (GLUT1) is highly expressed in cancer cells due to metabolic reprogramming^6^, whereas mannose receptors (MR) are typically overexpressed in tumor-associated macrophages (TAMs), which support tumor progression via anti-inflammatory mechanisms. Among glycosylation-based drug delivery methods, targeting overexpressed MRs using mannosylated carriers is one of the most reported strategies. The specificity and uptake of drugs by TAMs can be enhanced by conjugating delivery vehicles with mannose moieties. This approach enhances selective delivery to mannose receptor-expressing cells within the tumor microenvironment, while minimizing systemic toxicity and off-target effects. 7,8.

Albumin has also gained increasing recognition as a versatile nanoplatform because of its intrinsic biocompatibility, long circulation half-life, and ability to interact with multiple receptors, including gp60, FcRn, and SPARC. This unique combination of biological properties has driven extensive research into albumin-based nanomedicine platforms, highlighting their potential as clinically relevant drug carriers 9-12. Our previous study further demonstrated that SPARC mediates active albumin accumulation in glioma, providing direct evidence that albumin can serve as an actively targeted delivery system in tumors 13. In parallel, recent advances have expanded the versatility of albumin nanoplatforms, ranging from transformable nanocapsules that modulate tumor metabolism to albumin-hitchhiking immune conjugates that enhance tumor accumulation and potentiate antitumor responses 14,15. Together, these findings underscore the translational promise of modular albumin-based carriers.

For the glycosylation of drug delivery vehicles, direct conjugation methods involving amino groups or thiourea linkages have been used predominantly. However, because of the small size of sugar molecules and the nature of the conjugation methods, it is challenging to precisely control the number of sugar moieties attached to a single nanoparticle 3. To address this issue, we previously reported a clickable albumin nanoplatform (CAN) to produce various types of glycosylated albumin nanoparticles 16. CAN enables the precise regulation of the number of sugar molecules attached to each nanoparticle. This platform offers advantages owing to its scalability for large-scale production, which facilitates effective clinical translation 16,17. We leveraged this strategy to target various types of cells in the TME in this study.

An additional challenge is determining the primary cellular targets of glycosylated nanoplatforms* in vivo*. Tumors express numerous glycan-binding proteins, and glycosylated nanoparticles can interact with multiple cell-surface receptors, complicating the identification of ligand-receptor interactions 18,19. Spatial transcriptomics has recently emerged as a next-generation RNA analysis technique that maps the expression of all transcripts within tissue sections, enabling the exploration of underlying molecular markers in the TME 20-22. When combined with histological data and drug distribution images, spatial transcriptomics can be a powerful tool for drug discovery and TME research 23-25. Building upon our previous use of spatial transcriptomics to evaluate the lipid nanoparticle distribution in 4T1 tumor tissues 24, we adapted this method here to systematically identify cell types and molecular markers linked to the uptake of glycosylated albumin nanoplatforms.

In this study, we engineered a finely tuned albumin nanoplatform, referred to as Man-Alb, Glc-Alb, or Gal-Alb, depending on whether mannose, glucose, or galactose was incorporated. We hypothesized that these distinct glycosylations would result in unique biodistribution profiles and selective cell targeting within the TME, ultimately improving the therapeutic outcomes for the delivered drug.

## Methods

### Reagents and Experimental Equipment


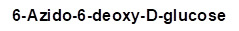
All chemicals were of reagent grade and were used without further purification. azadibenzocyclooctyne-NHS ester (ADIBO-NHS), 2,2',2''-(2-(4-(3-(3-azidopropyl)thioureido)benzyl)-1,4,7-triazonane-1,4,7 -triyl)triacetic acid (N_3_-NOTA), Flamma 648 azide (N_3_-FNR648) and Flamma 552 azide (N_3_-FNR552) were purchased from FutureChem Co., Ltd (Seoul, Korea). 1-*O*-(2-(2-(2-azidoethoxy)ethoxy)ethoxy)-alpha-D-mannopyranoside (alpha-Man-TEG-N3, Man-N_3_) and 1-O-(2-(2-(2-Azidoethoxy)ethoxy)ethoxy)-beta-D-galactopyranoside (beta-Gal-TEG-N_3_, Gal-N_3_) were purchased from Iris Biotech GmbH (Marktredwitz, Germany). 2-Azido-2-deoxy-D-glucose (Glc(2)-N_3_) and 6-Azido-6-deoxy-D-glucose (Glc(6)-N_3_) were purchased from Sigma-Aldrich (St. Louis, MO, USA). HSA was purchased from MP Biomedicals (Aurora, OH, USA). All other reagents and chemicals were purchased from Sigma-Aldrich (St. Louis, MO, USA). A size-exclusion PD-10 column was purchased from GE Healthcare Life Sciences (Buckinghamshire, UK). Instant thin-layer chromatography-silica gel (ITLC-SG) plates were purchased from Agilent Technologies, Inc. (Santa Clara, CA, USA). Radioactivity was measured using a gamma-scintillation counter (Packard Cobra II; GMI, NM, USA). The molecular weights of HSA and its conjugates were determined by MALDI-TOF/TOF mass spectrometry using the TOF/TOF 5800 system (AB Sciex, Foster City, CA, USA). The albumin concentration was spectrophotometrically measured using a NanoDrop® ND-1000 spectrophotometer (NanoDrop Technologies, Wilmington, DE, USA).

### Glycosylated Clickable Albumin Nanoplatform Synthesis & Characterization

The CAN preparation has been described previously. The procedure for large-scale synthesis was slightly modified to minimize batch-to-batch variation. Human Alb fraction V was purchased from MP Biomedicals (Aurora, Ohio, USA). Albumin was dissolved in phosphate-buffered saline (PBS) to a concentration of 250 mg/2.5 mL. ADIBO-NHS was dissolved in DMSO (20 mg/50 µL). Then, 10 µl of ADIBO-NHS solution were added to the albumin solution (0.5 mL), and four additional identical reaction sets were made (Sample 1-4). The molar ratio of albumin to ADIBO was 1:11. The amount of albumin used (50 mg, 1.5 mM) corresponds to 757.6 nmol, while the molar quantity of 4 mg of ADIBO was 8448.1 nmol (16.9 mM). The reaction was done at room temperature for 4 h with stirring, followed by overnight incubation at 4 °C. After the reaction, the mixture was filtered using a 30 kDa centrifugal filter. This involved centrifugation at 10,000 rpm for 3 min, repeated twice. The final volume is adjusted to the initial volume of 0.5 mL, followed by the recovery process. The resulting ADIBO-albumin was then diluted 40-fold for UV-vis absorbance measurements, and further analysis was conducted using MALDI-TOF to determine the molecular weight and DOF. The ADIBO-albumin conjugate was lyophilized for further experiments. For each study, the lyophilized intermediates were freshly reconstituted in PBS immediately prior to glycosylation with monosaccharides (glucose, mannose, or galactose) and subsequent use in *in vitro* and *in vivo* experiments.

For glycosylation, solutions of 5 mg/mL (14822 nmol/mL) Man-N_3_ and Gal-N_3_, and 3 mg/mL (14622 nmol/mL) Glc(2)-N_3_ and Glc(6)-N_3_. A total of 100 μL is extracted from the synthesized ADIBO-albumin solution, which has a concentration of 48.9 mg/0.5 mL (742.3 nmol/0.5 mL). To prepare Man-Alb, Gal-Alb, and Glc-Alb, add 50 μL each of Man-N_3_, Gal-N_3_, and Glc-N_3_ into the 100 μL of ADIBO-albumin. It is important to note that depending on the site of N_3_ introduction on the glucose molecule, Glc can form Glc(2)-Alb or Glc(6)-Alb. The ADIBO-albumin is referred to as simply "Albumin" in the paper. Notably, all the glycosylation reactions were conducted in a 1:5 molar ratio with respect to albumin, resulting in a final DOF of 4 for all glycosylation reactions. Similar to the previous ADIBO-albumin reaction, these glycosylation reactions also occur at room temperature followed by overnight incubation at 4 ℃. The purification process using a 30 kDa centrifugal filter is performed in the same manner, and the final volume is adjusted to the starting volume of 100 μL after recovery. Particularly, for experiments involving fluorescence, immediately after glycosylation, Flamma 648 azide (N_3_-FNR648) and Flamma 552 azide (N_3_-FNR552) were added to glycosylated albumin. Specifically, N_3_-FNR648 was introduced into Man-Alb, whereas N_3_-FNR552 was introduced into Gal- or Glc-Alb. The ratio of fluorescent reagents to glycosylated albumin was determined to be 1:10, indicating that the amount of fluorescent reagent was ten times less than the molar quantity of albumin. The reaction was conducted for a short duration of approximately 30 min, after which the same purification process was performed immediately.

### Radiolabeling Albumin by N_3_-NOTA

The vial containing ^64^Cu radioisotope was dried at 78 ℃ using a flow of dry nitrogen (N_2_) air. After the vial was completely dried, the pH was adjusted to 5 with 1 M sodium acetate buffer (pH 5.3, 100 µL). N_3_-NOTA solution (18 nmol/10 µL, dissolved in DW) was added and heated at 70 °C for 10 min. Radiolabeled N_3_-NOTA reacts with glycosylated albumin using a molar quantity of ^64^Cu labeled N_3_-NOTA which is ten times less than the amount of albumin. Additionally, it is essential to add an amount (volume) of radiolabeled N_3_-NOTA that is over 10 times smaller than the volume of the solvent (PBS) containing the reacting albumin. This ensured that the click reaction for albumin labeling could be performed without significant changes to the pH of the PBS solution. Radiolabeling efficiency was determined using radio-instant thin-layer chromatography-silica gel (radio-ITLC-SG) using 0.1 M citric acid as the mobile phase (Rf of radiolabeled N_3_-NOTA = 0.7-0.8; Rf of free isotope = 0.9-1.0; Rf of radiolabeled albumin conjugate = 0.0-0.1).

### Animal Model

The breast carcinoma cell line, 4T1 was cultured in RPMI-1640 medium supplemented with 10% fetal bovine serum (FBS) and 1% penicillin/streptomycin (PS). All cells were cultured at 37 °C in a humidified incubator containing 5% CO_2_. For tumor models, syngeneic BALB/c mice were used for 4T1 cells. Orthotopic tumor injections were performed by administering 4T1 cells (2 × 10^5^) to the right inguinal fourth mammary fat pads of 7-to 8-week-old female mice.

### PET Imaging of Different Glycosylated Clickable Albumin Nanoplatform

PET images were acquired using a preclinical PET/X-ray scanner (Sofie Bioscience) to confirm the pharmacokinetic parameters and biodistribution of different glycosylated albumin nanoplatform in normal and 4T1-bearing mice. Simultaneously, the same amount of each derivative (100 µg (1.5 nmol)/mouse) was intravenously injected into mice, and all images were acquired for 5 min at 0, 2, 4, and 24 h after injection. PET images were analyzed using the MIMvista software (MIM Software Inc., USA). The three-dimensional region of interest was used for the quantitative evaluation of uptake in the lungs, liver, and tumor (for the models).

### Biodistribution Analysis

The biodistribution of the different glycosylated albumin nanoplatform was evaluated in normal and 4T1-bearing mice. All mice were sacrificed in a CO_2_ chamber, 24 h after injection, and the heart, liver, lungs, spleen, stomach, kidneys, intestines, and tumors were carefully extracted. The radioactivity of each organ was measured using a gamma-scintillation counter. The results are expressed as %ID/g (injected dose per gram tissue weight).

### Targeting Study of Hepatocytes and Kupffer Cells in the Liver Using *Ex Vivo* Fluorescence Imaging

Man-Alb and Gal-Alb, each at a concentration of 100 μg/100 μL, are mixed and simultaneously injected into mice. Notably, Man-Alb was labeled with Flamma 648 azide fluorescence, whereas Gal-Alb was labeled with Flamma 552 azide. PET imaging-based time-activity graphs demonstrated that the maximum signal in the liver reached the 4-hour mark after intravenous injection of the albumin platform. This insight has informed the planning of a protocol for extracting tissues at the 4-hour mark post-injection. Importantly, due to the use of albumin platforms with pre-introduced fluorescence, the subsequent protocol involves slicing 4 μm sections of paraffin-embedded liver tissue without further steps of deparaffinization or hydration. The sections were blindly and randomly evaluated, and images were captured using a Leica TCS SP8 STED CW instrument (20×/0.7 numerical aperture objective lens) on a DMI 6000 inverted microscope (Leica, Mannheim, Germany) and MetaMorph version 7.8.10 software (Universal Imaging, Downingtown, PA, USA). FNR648 had an excitation wavelength of 648 nm and an emission wavelength of 672 nm. For the fluorescence equipment, the excitation wavelength was set to 620 nm, while the emission wavelength was set to 670 nm. The fluorescence characteristics of FNR552 are similar to those of Cy3; therefore, the settings for tissue fluorescence imaging were configured according to the wavelength specifications for Cy3.

### ST Library Acquisition

Two 4T1 tumor-bearing mice were prepared. Different albumin nanoplatforms were administered through the tail veins of the mice. For the Man+Glu sample, a mixed solution of Flamma Fluors 648 labeled mannosylated and Flamma Fluors 552 labeled glucosylated albumins was administered. Moreover, for the Man+Gal sample, a mixed solution of Flamma Fluors 648 labeled mannosylated albumins and Flamma Fluors 552 labeled galactosylated albumins was administered. After acquiring two optimal cutting temperature (OCT) compounds embedded in fresh frozen tumor samples, three adjacent tumor sections (one for hematoxylin and eosin (H&E) staining, another for fluorescence (FL) imaging, and the other for ST library) for each sample were obtained. Formalin-fixed, paraffin-embedded (FFPE) sections were not considered, as paraformaldehyde for FFPE was not suitable for FL imaging, other than methanol for FF. ST libraries were acquired using the Illumina HiSeq platform. While running SpaceRanger on the raw ST library, mm10 (Mus musculus) was used as the reference genome.

### Fluorescence Image Processing and SPADE Algorithm

For each Visium ST library, the red or green fluorescence value mapped to each spot was divided by the total value across all spots in the library and then multiplied by 100 to normalize the fluorescence intensity. The normalized fluorescence for each spot indicated the proportion of albumin nanoparticles delivered to that spot relative to the total in the tissue corresponding to the Visium library. Normalized intensities were depicted differently using an R ggplot for each spatial cluster and glycosylation. A separate p-value (t-test) was used to compare the two platforms in each cluster.

Our team developed an algorithm called spatial gene expression patterns using deep learning of tissue images (SPADE) to connect FL images to the corresponding ST library 23. Using the SPADE algorithm, each spot can have a 512D feature vector per FL image, resulting in 512 mapping images. For subsequent analyses, we utilized only the mapping image that had the most variable principal components from the 512D feature vectors. This was considered the most representative fluorescence (FL) mapping profile for the ST library.

### Differentially Expressed Gene (DEG) Exploration

The most significant DEGs were examined by sorting fold-change (FC) values and exclusively concentrating on genes with an adjusted p-value of less than 0.05. When drawing GO plots, 20 mouse genes were considered when drawing the GO plots. However, when performing IAMSAM, all genes satisfying the given criteria were input to acquire the GO plots.

### CellDART

To acquire cell-type distribution in ST libraries, a cell-type deconvolution algorithm called cell-type inference by domain adaptation of single-cell and spatial transcriptomic data (CellDART) was introduced 26. We used publicly available 4T1 data 27 as a single-cell RNA sequencing (scRNA-seq) reference to execute the CellDART algorithm. Originally, nine cell types were included in the scRNA-seq reference: inflammatory macrophages (M1 cells), endothelial cells, neutrophils, proliferative myeloid cells, monocyte-derived cells, fibroblasts, epithelial cancer cells, T cells, natural killer cells, and anti-inflammatory macrophages (M2 cells). To differentiate TAM-like M2 cells from non-TAM-like M2 cells in anti-inflammatory macrophages, approximately half of the anti-inflammatory macrophages expressing previously reported TAM markers 28, Folr2, C1qa, C1qc, C1qb, Ccl8, Ccl2, Siglec1, Tcn2, Ccl4, Hcst, Pltp, Lap3, Tnfaip3, Ccl3, Dok2, Cd83, and Aif1 were separated from other M2 cells.

### IAMSAM

To investigate the gene signatures for ROIs, we introduced an image-based analysis of molecular signatures using the developed segment-anything model (IAMSAM). The prompt mode was used to pinpoint the specific uptake patterns of interest. Otherwise, we followed the general running protocol. To acquire GO plots, a log FC cutoff of 1 and an adjusted p-value cutoff of 0.05 were applied.

### Micro-distribution of Man-Alb in Tumor Tissues

Man-alb with Flamma Fluors 648 (1 mg/0.1 mL PBS) was injected into 4T1 tumor-bearing mice, and tumor tissues were excised 24 h after injection. The tissues were fixed in 4% paraformaldehyde for at least 24 h and sectioned at 4 μm thickness.

Paraffin-embedded sections were deparaffinized, hydrated, and subjected to antigen retrieval by steaming in sodium citrate buffer (10 mM sodium citrate, pH 9.0). After blocking with a solution containing 10% FBS and 1% serum albumin in TBS, the slides were incubated overnight at 4 ºC with an anti-CD206 antibody (1:1000, ab64693, Abcam). Biotinylated secondary antibodies were applied, and signal development was carried out using liquid 3,3'-Diaminobenzidine (DAB) substrate (Dako, Glostrup, Denmark) to achieve the desired brown color. The sections were counterstained with H&E (Abcam). Slides were scanned using a digital camera (Aperio AT2; Leica, Wetzlar, Germany) at 100× magnification.

Serial deparaffinized and hydrated sections were counterstained with 1 mg/mL DAPI solution (Sigma-Aldrich). Slides were scanned using a Stellaris 8 confocal fluorescence microscope (Leica, Wetzlar, Germany).

### Preparation of Clodronate-loaded Glycosylated Albumin

For the drug-loading process, disodium clodronate tetrahydrate (4 mg/0.2 mL) was dissolved in PBS and mixed with glycosylated albumin (20 mg/0.5 mL) at a molar ratio of 1:36. The mixtures were incubated overnight at room temperature. Subsequently, the mixture was purified twice by centrifugation at 10,000 rpm for 3 min to obtain the clodronate-loaded albumin complex. The free drug in the filtrate was quantified by measuring the absorbance at 205 nm using a UV-vis spectrophotometer. A standard calibration curve was used to calculate the drug-loading efficiency (LE) of albumin, as follows:

LE (%) = [(Total amount of drug - Free drug in the filtrate) / Total amount of drug] × 100

To examine the drug release kinetics, the samples (clodronate dose: 400 µg/mL) were diluted in 5 mL of PBS and incubated at room temperature. At predetermined time points (0, 2, 4, 8, 16, 24, and 48 h), the solution (0.5 mL) was withdrawn and the amount of drug released from the samples was determined by UV-visible spectrophotometry. Cumulative release was calculated using the following equation:

Cumulative drug release (%) = (Amount of drug in the release medium / Initial amount of drug loaded on albumin) × 100

### Flow Cytometry

Mammary tumor tissue samples were mechanically dissociated to generate single-cell suspensions. Thereafter, erythrocytes were removed, and the resulting single-cell suspensions were incubated with purified anti-CD16/CD32 (BioLegend) for 15 min at 4 °C, and finally processed for cell-surface staining with the appropriate antibodies at 4 °C. The following antibodies were used: CD45 FITC (30-F11), CD11b V450 (M1/70), Ly6C PerCP-Cy5.5 (HK1.4), and CD206 PE (MR6F3) from eBioscience; and F4/80 PE-Cy7 (BM8) and Ly6G BV711 (1A8) from BioLegend. Data were acquired using the LSR Fortessa system (BD Biosciences) and analyzed with the FlowJo software (Tree Star, OR, USA).

### Statistics

R (ver. 4.0.5) and Python (ver. 3.7.12) were used as the programming languages. In addition, the Seurat (ver. 4.0.2), Scanpy (ver. 1.9.1), and SpaceRanger (ver. 2.0.1) were used for subsequent analyses. Parameters for Seurat in R were set to default except for SCTransform (variable.features.n = 1000), RunPCA (assay = “SCT”), FindNeighbors (dims = 1:30), RunTSNE (dims = 1:30), and topTable (adjust = “fdr”). The parameters for Scanpy in Python were set to default, except for scanpy.tl.pca (svd_solver = arpack), scanpy.pp.neighbors (n_neighbors = 10, n_pcs = 40), scanpy.tl.leiden (resolution = 0.5), and scanpy.pl.rank_genes_groups (n_genes = 40).

### Transmission Electron Microscopy (TEM) Analysis

Transmission electron microscopy (TEM) of glycosylated albumins (HSA, ADIBO-HSA, Glc-Alb, Man-Alb, Gal-Alb) was performed using a JEM-1400 instrument (JEOL, Japan). Samples were prepared in the concentration range of 5-20 mg/mL, and 20 μL of each sample was spotted twice onto 200-mesh carbon-coated copper grids. Excess liquid was blotted with filter paper, and the grids were air-dried for approximately 4 h prior to imaging. TEM observations were carried out at an accelerating voltage of 80 kV with a magnification of 120,000×.

### Dynamic Light Scattering (DLS) and ζ-potential Analysis

The hydrodynamic diameter of glycosylated albumins was measured using a Zetasizer Nano ZS90 system (Malvern Instruments, UK) at 25 °C. For DLS measurements, samples were prepared at a concentration of 20 mg/mL in PBS. For ζ-potential analysis, the same samples were diluted 100-fold in PBS prior to measurement.

## Results

### Albumin Nanoparticle Synthesis and Characterization

**Figure 1:**
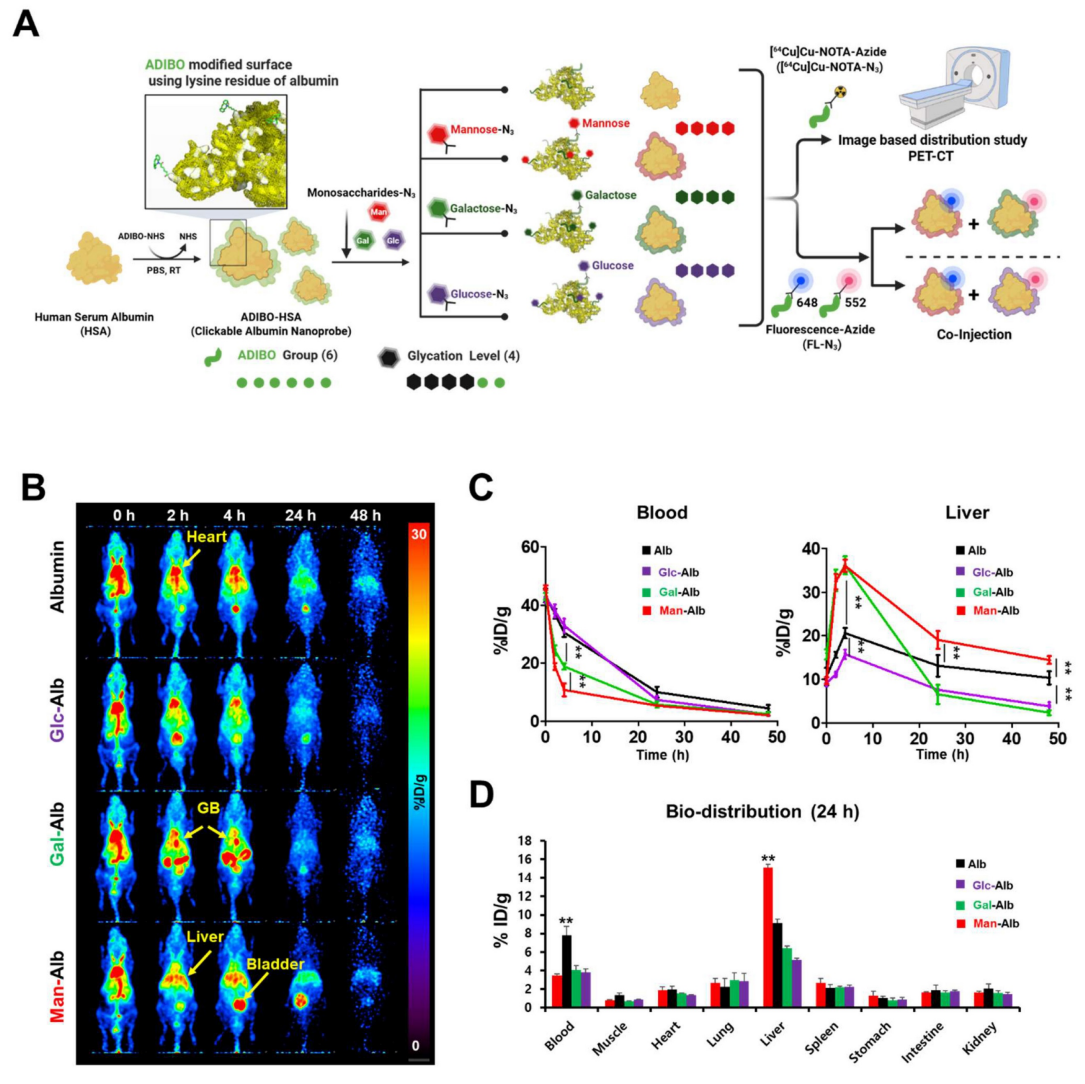
** Construction flow of the nanoplatform for this study and the validation of distinct glycosylation based on positron emission tomography imaging.** (A) Schematic flow of CAN-DGIT for confirming the efficient targeting property. (B) Representative PET images of ^64^Cu labeled distinct glycosylated albumin in normal mice following IV administration at various time points (0, 2, 4, 24, and 48 h; n = 4 for each group). The yellow arrow indicates the gallbladder (GB). (C) Time-activity curve of glycosylated albumin in blood and liver. (D) *Ex vivo* biodistribution of glycosylated albumin at 24 h after injection. All quantification was presented as %ID/g ± SD (**: P < 0.001).

Based on the azadibenzocyclooctyne (ADIBO)-functionalized CAN previously reported by our group^16^, we synthesized Man-Alb, Gal-Alb, and Glc-Alb, naming them clickable albumin nanoplatform engineered by distinct glycosylation for selective *in vivo* cell targeting (CAN-DGIT). As shown in **Figure 1A**, this process involves the reaction of azadibenzocyclooctyne-N-hydroxysuccinimide (ADIBO-NHS) with lysine residues on the albumin surface. This led to albumin formation with six ADIBO groups (AD^6^-Alb), and glycosylation was performed through click reactions to produce albumin molecules with four sugar moieties. The structures of the sugar azides and their respective molecular weights are shown (**Figure S1**). During this process, the increase in molecular weight based on reaction ratios, number of attached ADIBOs, and degree of functionalization (DOF) was calculated, as verified using MALDI-TOF-based DOF analysis (**Figure S2 and Table S1**). Additionally, the UV-visible spectrum demonstrated an increase in the peak intensities at specific wavelengths for both albumin (peak intensity at 280 nm, denoted by a black square box) and ADIBO (peak intensity at 309 nm, denoted by a blue square box), per the reaction ratio (**Figure S3 and Table S2**). All experiments were repeated at least 4 times.

To optimize the number of sugar moieties on CAN, we introduced azide-functionalized mannose (Man-N_3_), galactose (Gal-N_3_), and glucose (Glc-N_3_) into AD^6^-Alb at reaction ratios in a 5-fold excess. Subsequently, we prepared distinct glycosylated CANs with three different sugars, denoted as Man-Alb, Gal-Alb, and Glc-Alb. Hereafter, AD^6^-Alb is referred to as albumin in this study. In the glucosylation case, the numbers in parentheses indicate the carbon position of the azide introduced into the glucose moiety (Glc(2) or Glc(6)). Molecular weight analysis using MALDI-TOF confirmed the number of introduced carbohydrate moieties (**Table S3**). Subsequently, depending on the intended application, we labeled them with radioisotopes or fluorescent dyes for further use.

### Positron Emission Tomography (PET) Imaging-Based Distribution Study of CAN-DGIT

To verify the *in vivo* behavior of each glycosylated albumin nanoparticle and to determine whether different monosaccharide conjugations lead to distinct biodistribution patterns, we conducted a comprehensive study using positron emission tomography (PET) imaging. First, each albumin nanoparticle (Alb, Man-Alb, Gal-Alb, Glc-Alb) was labeled with ^64^Cu, allowing for real-time, quantitative tracking of the nanoparticles in healthy mice. We aimed to capture both the pharmacokinetic profiles (blood circulation and clearance) and organ-specific accumulation of each glycosylated nanoparticle.

**Figure 1B** shows representative PET images from 0 to 24 h post-injection. Notably, albumin (Alb) and Glc-Alb displayed similar blood-pool pharmacokinetics, maintaining relatively higher signals in circulation during the early time points. In contrast, Man-Alb and Gal-Alb were rapidly cleared from the blood pool and promptly taken up by the liver (**Figure 1C, Table S4**). This immediate high liver uptake likely reflects receptor-mediated interactions in hepatic tissues, emphasizing the role of specific glycan-protein binding events. Particularly, Gal-Alb demonstrated prominent localization in the gallbladder (yellow arrow) shortly after hepatic uptake, followed by a clear pattern of hepatobiliary excretion into the intestine. Man-Alb, however, exhibited limited excretion into the intestine and remained mostly within the liver, suggesting a distinct clearance mechanism for mannose-modified nanoparticles. These divergent pathways became especially evident between 2 and 4 h post-injection, where the PET signal for Gal-Alb moved from the liver into the gastrointestinal tract. By 24 h, Gal-Alb's overall residual signal was markedly reduced, indicating rapid *in vivo* clearance (**Figure 1B**). This characteristic can be advantageous for tumor-targeting applications since increased clearance from healthy tissues may enhance the tumor-to-organ ratio of the nanoparticle. In contrast, Man-Alb's persistent liver localization at late time points underscores the potential for prolonged hepatic retention, mediated by mannose receptors on Kupffer cells. To further quantify these observations, we performed *ex vivo* gamma counting of major organs at 24 h post-injection (**Figure 1D, Table S5**). The *ex vivo* biodistribution results were consistent with the PET data: albumin and Glc-Alb retained higher levels in the blood, whereas Man-Alb showed predominantly hepatic retention. The comparatively low residual signal of Gal-Alb highlights its relatively rapid elimination via the hepatobiliary route. These findings, taken together, establish that fine-tuning glycosylation on albumin nanoparticles significantly influences their pharmacokinetics and organ-specific distribution, even when the underlying nanoparticle core (albumin) is otherwise identical.

Considering these initial results, we selected Man-Alb and Gal-Alb as strong contrast examples for further investigation, focusing on the possibility that mannose-based modifications might predominantly target liver-resident cells (e.g., Kupffer cells), while galactose-based modifications could show a faster clearance pathway—potentially aiding tumor selectivity by minimizing prolonged liver accumulation. To examine this more closely, we planned a series of co-injection experiments using differently fluorescently labeled Man-Alb and Gal-Alb in the same mouse. This approach was chosen to minimize inter-animal variability and to clarify the specific cellular mechanisms (e.g., Kupffer cell vs. hepatocyte uptake) driving the differences in excretion pathways and overall biodistribution. The details of these fluorescence-based studies, as well as the accompanying spatial transcriptomics analyses, are described in the following sections.

### Comparison of Man-Alb vs Gal-Alb via *in vivo and ex vivo* imaging

**Figure 2:**
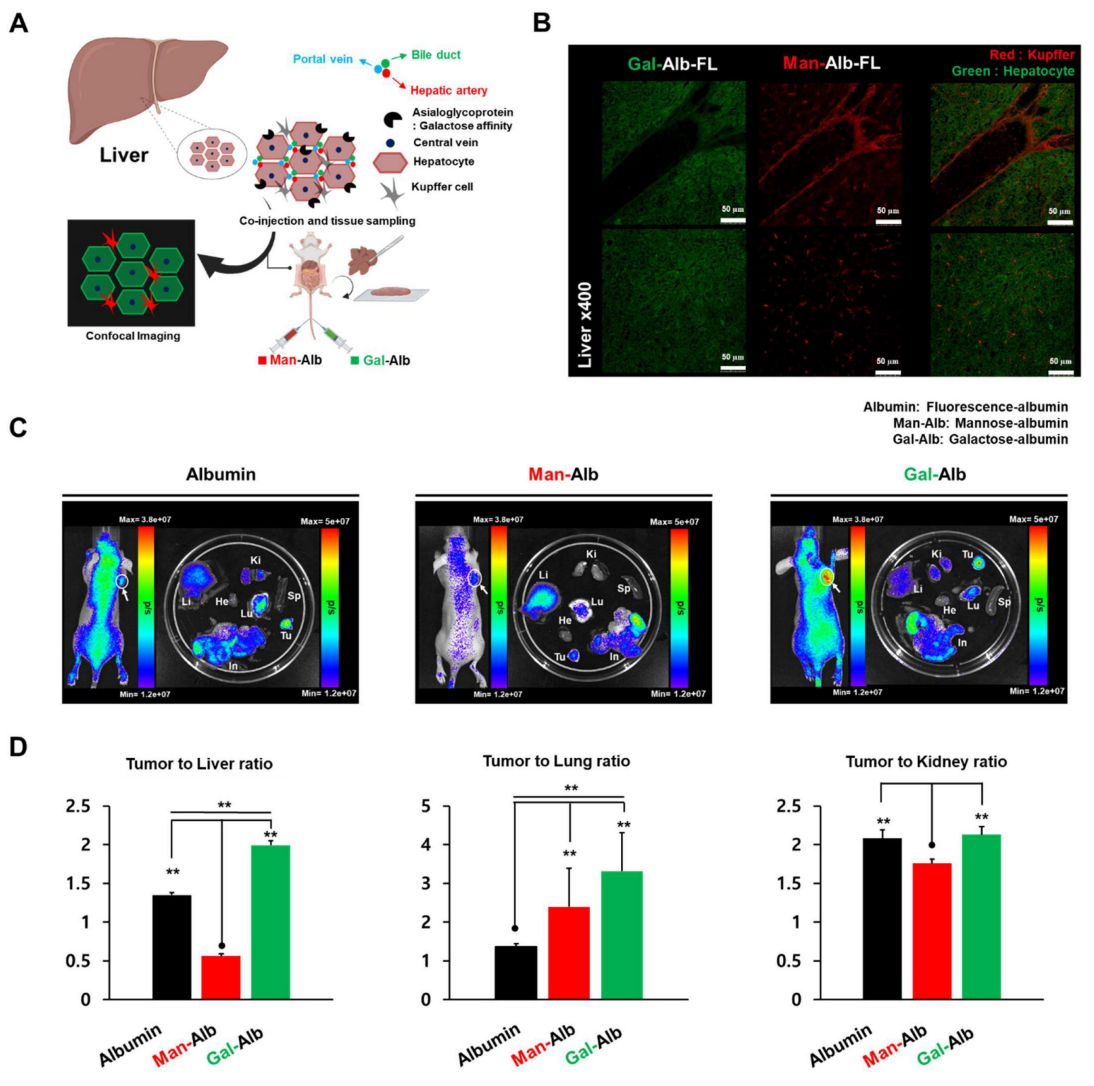
** Micro-specificity of mannose to Kupffer cell and galactose to hepatocytes in liver tissue**. (A) After simultaneous vascular injection of Man-Alb and Gal-Alb, liver tissue fluorescence imaging acquisition. Schematic figure of targeting for hepatocytes and Kupffer cells. (B) *Ex vivo* confocal Imaging of Man, Gal-Alb with different fluorescence filters. Upper panel: depicts the periportal region, emphasizing Kupffer cells enriched in the sinusoidal area adjacent to the portal venule. Lower panel: illustrates the liver parenchyma showing Kupffer cells residing within the sinusoidal lumen along hepatocytes. Flamma fluor 648-conjugated mannose albumin and Flamma Fluor 488-conjugated galactose albumin nanoparticles are scanned with appropriate ex/em filters (Red = Flamma fluor 648, Green = Flamma Fluor 488). (C) IVIS imaging of each type of glycosylated albumin after 24 IV injections. (D) Tumor-to-organ ratio of image-based quantification using IVIS. Abbreviations: Li, liver; Ki, kidney; Tu, tumor; He, heart; Lu, lung; Sp, spleen; In, intestine. Error bars represent mean ± SD.

To validate the different cellular targeting abilities of Man-Alb and Gal-Alb in the liver, we labeled Man-Alb with Flamma Fluor 648 and Gal-Alb with Flamma Fluor 552. Equal amounts of these labeled albumin platforms were co-injected into normal mice for *in vivo* imaging to confirm the differences at the cellular level **(Figure 2A)**. Given that the most distinct distribution difference was observed at 4 h in the image-based evaluation, we decided to sacrifice the mice at the 4-h time point to obtain liver tissues. Subsequently, we examined the different distributions at the cellular level by confocal microscopy. Interestingly, Man-Alb and Gal-Alb demonstrated substantially different microscopic localizations in the liver **(Figure 2B, Figure S4).** Upon reviewing the microscopic fluorescent images, we observed that Man-Alb precisely matched the histological localization of Kupffer cells, while Gal-Alb matched with hepatocytes 29. Immunostaining with CD206 (Kupffer cells) and ASGPR (hepatocytes) confirmed these distinct distributions (**Figure S5**), indicating that glycosylation type directs selective uptake by different liver cell populations.

### Analysis of Targeting Using the Fluorescence of Man-Alb and Gal-Alb in the Cancer Model

After confirming the different biodistribution and cell-targeting abilities in the liver of normal mice, IVIS imaging was performed in 4T1 tumor-bearing mice to further investigate and compare the biodistribution and tumor-targeting ability of Man-Alb, Gal-Alb, and Alb. For each albumin platform, a fluorescent dye (Flamma Fluors 648) was introduced in equal amounts and injected into 4T1 tumor-bearing mice. At 24 h after injection, *in vivo* and *ex vivo* fluorescence imaging were performed using IVIS (**Figure 2C-D**). In *ex vivo* imaging of the organs, liver Man-Alb uptake was most prominent, which is in line with PET imaging. Notably, Gal-Alb demonstrated the highest tumor-to-liver ratio (1.99 ± 0.06), while Man-Alb demonstrated the lowest (0.56 ± 0.02; **Figure S6 and Table S6**). This was caused by the low liver uptake of Gal-Alb at a late time point, caused by rapid clearance through hepatobiliary excretion (**Figure 1**). When comparing tumors to lungs, albumin had the lowest ratio, which could be attributed to the high amount of residual blood observed at 24 h in PET imaging. This approach may help prevent sustained liver uptake and the associated liver toxicity, which are significant concerns in traditional nanoparticle-based strategies. Additionally, this result indicates that galactosylation of nanoparticles may enhance their tumor-to-liver uptake ratio, thereby broadening their therapeutic windows 30.

### Spatial Clustering Analysis Reveals Distinct Uptake Patterns of Man-Alb and Gal-Alb

**Figure 3:**
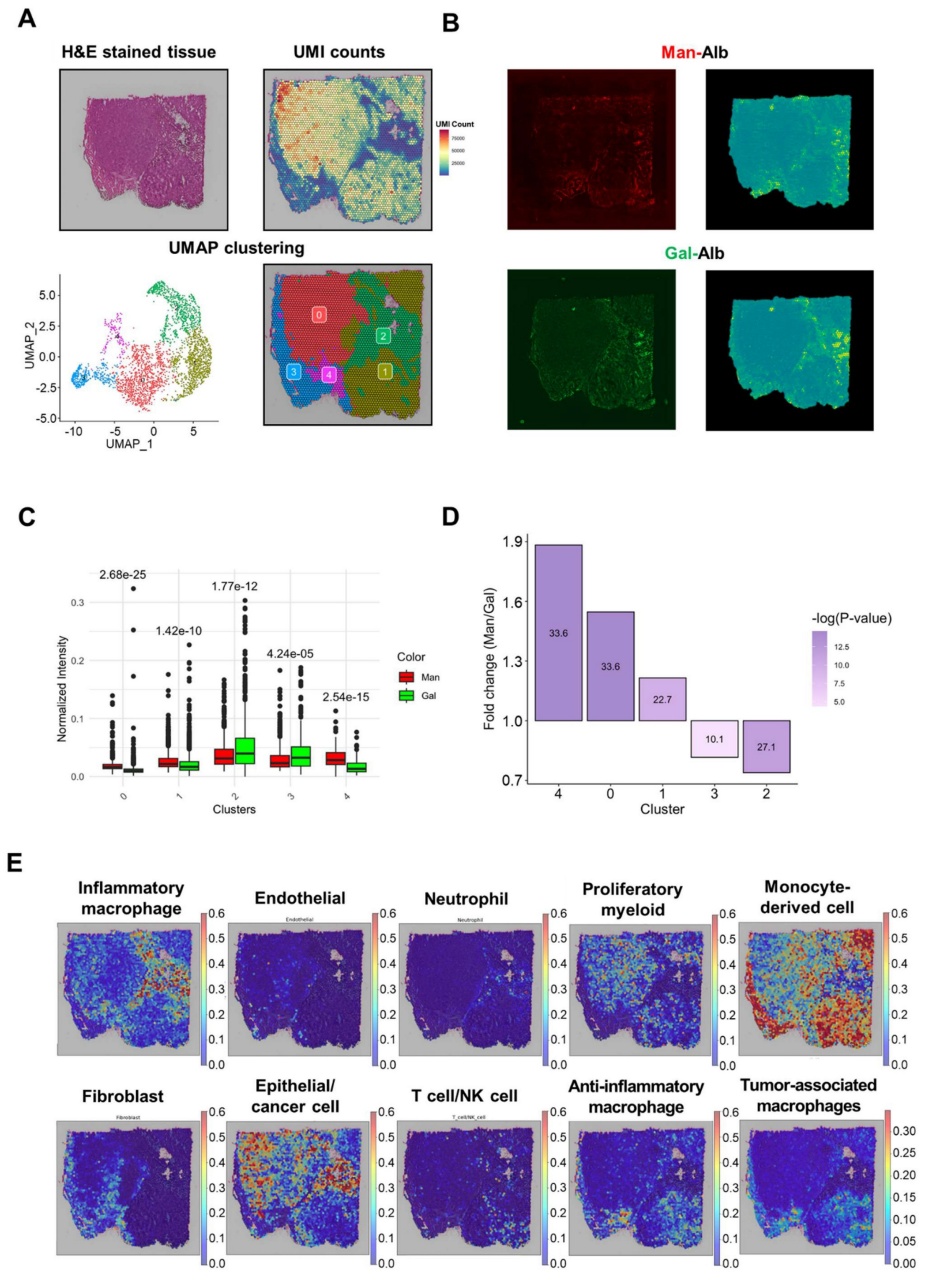
** Summary of ST library of Man+Gal sample.** (A) The images were derived from spatial transcriptomic (ST) data of the Man+Gal sample. Each image represents the H&E image (upper left), the UMI counts provided by SpaceRanger (upper left), and the spatial clustering analysis of the Man+Gal sample (below). UMAP projections of spatial clusters after data integration with the Man+Gal sample by using the Seurat pipeline (left) and the distribution of spatial clusters according to sample (right). (B) Fluorescence images of Man+Gal sample. Flamma fluor 648-conjugated mannose albumin and Flamma Fluor 488-conjugated galactose albumin nanoparticles are scanned with appropriate ex/em filters (left). Additionally, they mapped with ST spots using the SPADE algorithm (right). (C) Relative fluorescence signals of each cluster mapped by SPADE algorithm to ST libraries according to the albumin nanoplatform. Error bars represent mean ± SD. (D) Mean intensities of min-max normalized albumin fluorescence signals in each cluster. P-values between mannose and galactose albumin fluorescence signals. (E) CellDART results for the Man+Gal sample. The original 4T1 scRNA-seq reference for CellDART execution contained only nine cell types, among which the original 'Anti-inflammatory_macrophages' was divided into the newly defined 'Anti-inflammatory_macrophages' and 'Tumor-associated_macrophages' for non-TAM-like cells and TAM-like cells, respectively.

Because tumors are more heterogeneous than the liver, we employed spatial transcriptomics (ST) to delineate how Man-Alb and Gal-Alb distribute among various tumor cell types. Different fluorescent labels for the two nanoparticles were co-injected into 4T1-bearing mice, followed by ST analysis of tumor sections. To address potential batch effects, we integrated data from multiple libraries using the Seurat pipeline (**Figure 3A**). We then applied the SPADE algorithm 23 to map fluorescence (FL) signals to ST spots, reducing image noise via principal component filtering (**Figure 3B**).

### Integrative Analysis of Clusters and FL Signals Reveals Distinct Uptake Patterns Between Man-Alb and Gal-Alb

When exploring the DEGs and gene ontology (GO) for each spatial cluster, unique biological activities were observed (**Figure 3A, Figure S7, Tables S7-11**). In clusters 0 and 1, where albumin platforms rarely appeared, extracellular matrix and neutrophil gene signatures were noted. Additionally, in cluster 2, where Gal-Alb appeared more frequently than Man-Alb, specific metabolisms, including '*cellular response to metal ion,*' and '*cellular response to cadmium ion*' appeared, implying active metabolism. Active material metabolism may be linked to increased Gal-Alb uptake.

The FL signal of each albumin platform is predominantly concentrated within a few spatial clusters. For example, Man-Alb uptake was higher in cluster 4 than in clusters 0 and 1. When comparing the relative FL signals between Man-Alb and Gal-Alb, Man-Alb appeared relatively frequently in cluster 4, whereas Gal-Alb appeared in cluster 2 (**Figure 3A, B).** Moreover, when normalizing the Man-Alb and Gal-Alb fluorescence signals by min-max normalization, the mean fluorescence intensities of each cluster showed different trends for Man-Alb and Gal-Alb. In clusters 0, 1, and 4, the mean Man-Alb intensities were higher than those of Gal-Alb. However, in clusters 2 and 3, the mean Gal-Alb intensities were significantly higher than those of Man-Alb. In cluster 4, Man-Alb uptake was approximately 1.883 times higher than Gal-Alb uptake, whereas, in cluster 2, Man-Alb uptake was approximately 0.739 times lower (**Figure 3C, D**), suggesting that a targeting process may occur in cluster 4 for Man-Alb and in cluster 2 for Gal-Alb.

### Specific TME Cell Types Associated with Man-Alb and Gal-Alb

The associated cell types in each cluster were explored using the cell-type deconvolution algorithm, CellDART 26 (**Figure 3E, Figure S8**). Cancer epithelial cells and inflammatory macrophages were predominantly observed in spatial cluster 2.

The active metabolism shown in the GO analysis of the cluster was found to be active cancer-specific metabolism (**Figure S7**). Anti-inflammatory macrophages and fibroblasts were in cluster 4, an extracellular matrix-associated cluster. This can be explained by previous research suggesting that the ECM and collagen influence the polarization and promotion of macrophages^31^,^32^. In addition, tumor-associated-like macrophages (TAM-like M2 cells) showed a starker appearance than non-TAM-like M2 cells and concurred in cluster 4, where the Man-Alb distribution was dominant. Moreover, when we performed a correlation analysis between CellDART results and fluorescence intensities, anti-inflammatory macrophages and TAMs demonstrated a weak positive correlation with Man-Alb uptake in the whole tissue (**Table S12**). When the same analysis was performed for each cluster, cluster 3, with a small Man-Alb signal and TAMs distribution region, showed similar results (**Tables S13-17**). Thus, Man-Alb uptake may be influenced by TAM-like M2 cells, whereas Gal-Alb uptake may be influenced by inflammatory macrophages and cancer epithelial cells.

**Figure 4:**
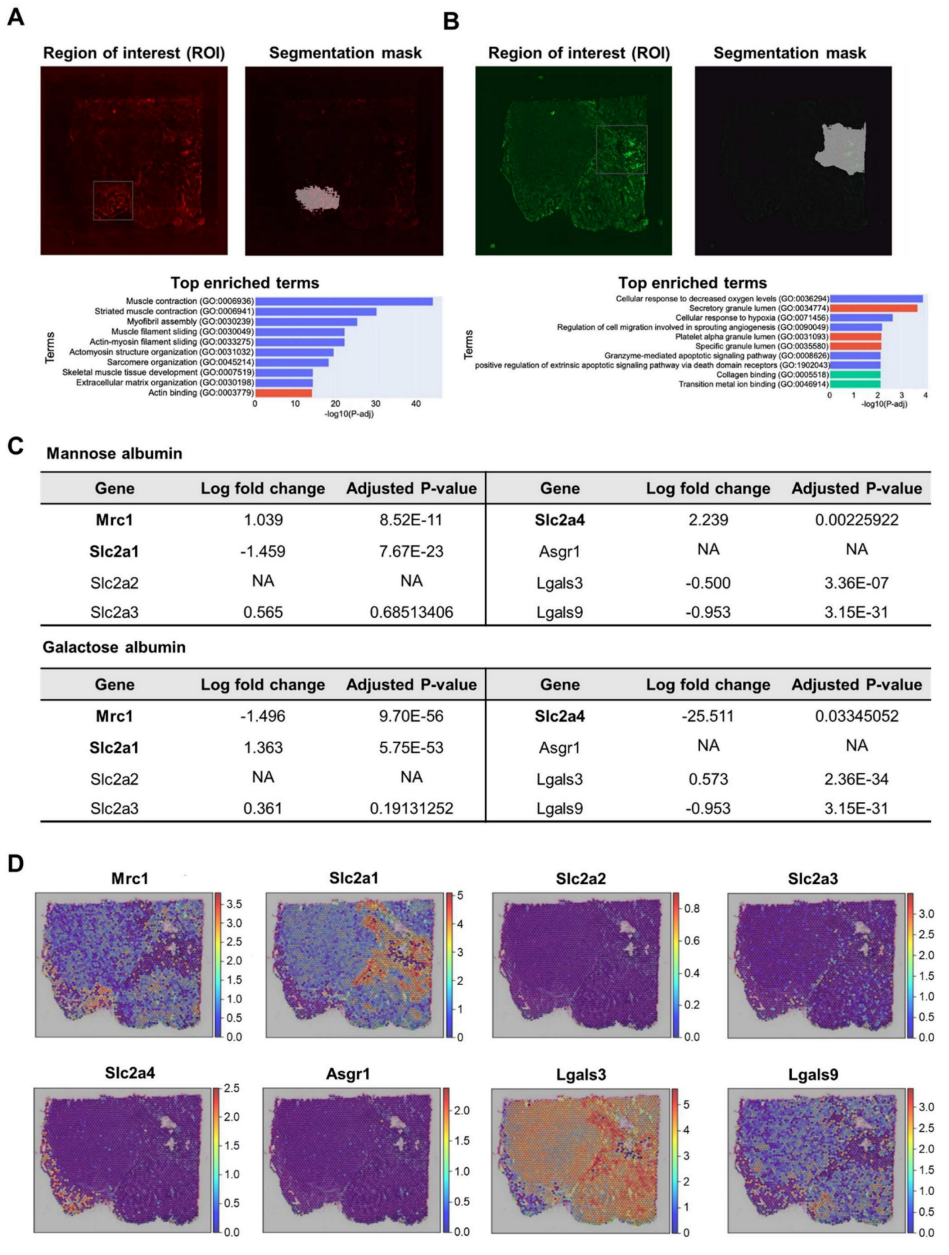
** Analysis of high CAN-DGIT uptake regions of Man+Gal sample.** (A) IAMSAM analysis of relatively high mannose albumin uptake region. The characteristic uptake region of mannose albumin was pointed as a gray rectangular box (upper left). When this region is applied in a segment-anything model, distinct albumin uptake patterns in the region are segmented (upper right). Additionally, differentially expressed genes in this ROI were listed and used for gene set enrichment analysis (below). (B) The same analysis was performed in the galactose albumin distribution image. (C) Table of log fold change and adjusted p-values for comparing gene expression levels of each glycan-related gene between segmented ROI from IAMSAM and the rest region. (D) Expressions of mannose receptor, glucose transporter, and asialoglycoprotein receptor-related genes.

To identify the biological processes, including glycan-binding proteins, sugar receptor expression was involved in the uptake of glycosylated albumins in regions of interest (ROI), which are distinctive uptake regions of Man-Alb or Gal-Alb, we performed semi-automatic segmentation of images (IAMSAM) 33. For the Man-Alb platforms, the expected stromal activities were observed, consistent with their increased uptake in cluster 4 (**Figure 4A, Table S18**). Moreover, during the analysis of the Gal-Alb platforms, glycolytic and hypoxia genes were observed (**Figure 4B, Table S19**). This implied that the Gal-Alb uptake was cancer cell-driven, as expected from the CellDART results. In addition, when exploring the expression levels of mannose-, glucose-, and galactose-related transporter and receptor genes in the ROIs, mannose receptor C-type 1 (Mrc1), solute carrier family 2 member 1 (Slc2a1), and solute carrier family 2 member 4 (Slc2a4) genes were differentially expressed (|log_2_ fold change| > 1, P < 0.05; **Figure 4C**).

In the Man-Alb uptake-distinct region, which was similar to cluster 4, Mrc1 and Slc2a4 were more highly expressed than in the other regions, whereas Slc2a1 was less expressed than in the other regions. Finally, the spatial expression patterns of Mrc1 and Slc2a1 explained the uptake of Man-Alb and Gal-Alb (**Figure 4D**). It is particularly relevant that the M2 macrophages actively utilize MR (C-type lectin receptors) to obtain extracellular ligands. Moreover, cancer cells preferentially located in cluster 2 may affect the uptake of other albumins because various receptors (*e.g.*, Slc2a1, glucose transporter 1 gene) are expressed on the cell membrane to actively collect ligands. Gal-Alb uptake did not show concurrent results with the asialoglycoprotein receptor 1 (Asgr1) and galectin 9 (Lgals9) genes, implying that their uptake may be receptor-mediated by Slc2a1 and galectin 3 (Lgals3), or active-metabolism-driven by cancer cells.

By applying the same analysis to genes encoding glycan-binding proteins, we observed that in regions with high Man-Alb intake, the mannose receptor C-type 2 (Mrc2) gene and selectin P (Selp) were overexpressed. Moreover, overexpression of glycan-binding-related genes, such as C-type Lectin Domain Family 11 Member A (Clec11a), C-type Lectin Domain Family 2 Member D (Clec2d), and C-type Lectin Domain Family 3 Member B (Clec3b), was notable. In contrast, in regions with predominant Gal-Alb intake, overexpression of galactose-binding-related genes, such as Lgals3 and Galectin 7 (Lgals7), was noted (**Tables S20, S21, Figure S9**).

### Comparison Study of Man-Alb and Glc-Alb Based on PET Imaging

**Figure 5:**
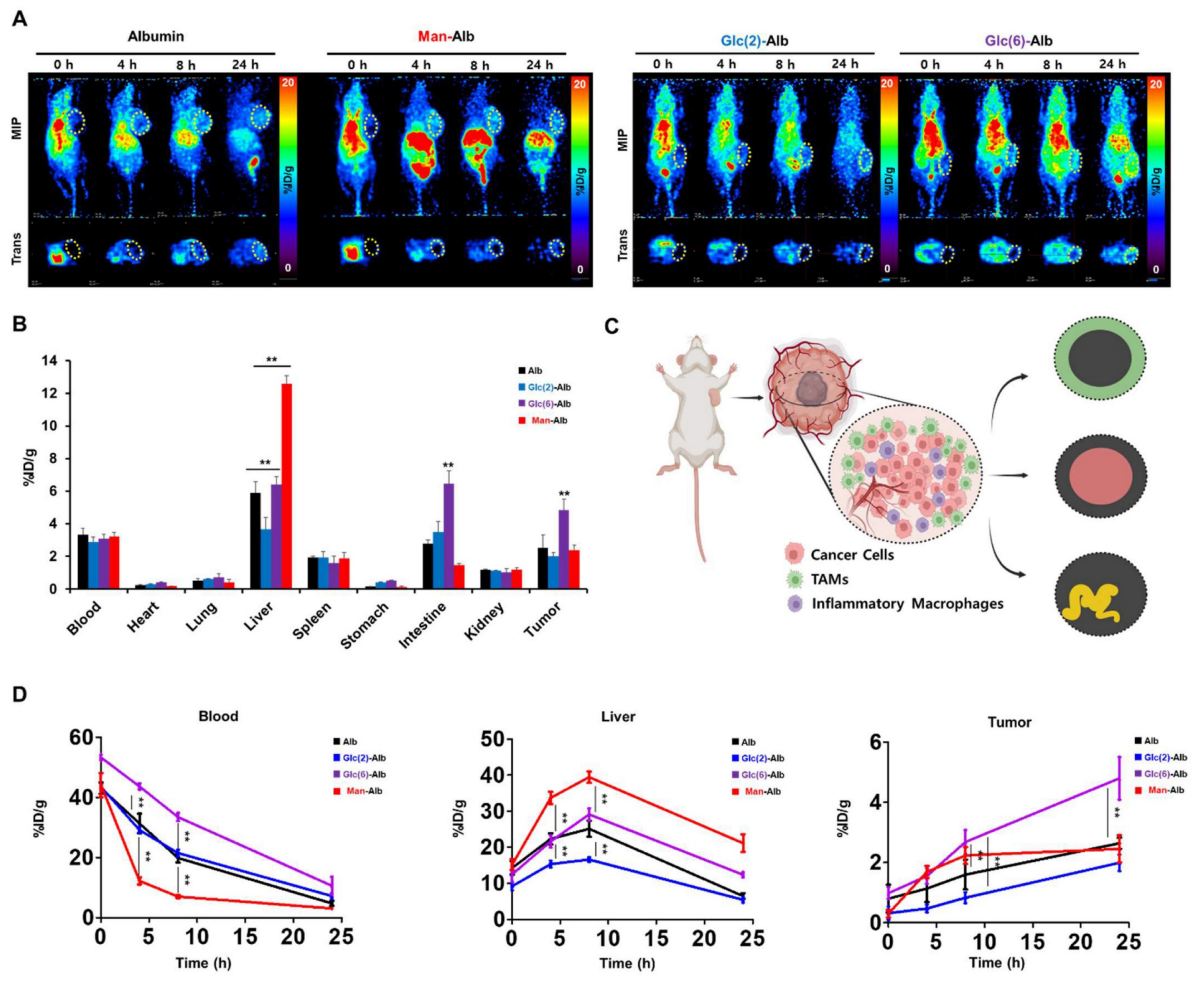
***In vivo* and *ex vivo* biodistribution of ^64^Cu radiolabeled distinct glycosylated albumins in the 4T1 tumor model.** (A) Representative PET imaging of 4T1-bearing mice using Albumin, Man-Alb, Glc(2)-Alb and Glc(6)-Alb following IV administration at various time points (0, 4, 8, and 24 h; n = 4 for each group). (B) *Ex vivo* biodistribution of ^64^Cu radiolabeled distinct glycosylated albumins in tumor models measured using a gamma counter at 24 h after injection (n = 4 for each group). (C) Representative illustration of *in vivo* cell targeting in cancer region. The cells highlighted in red represent cancer cells, the green ones are tumor-associated macrophages (TAMs), and the purple ones indicate inflammatory macrophages. D. Time-activity curve of glycosylated albumin in blood, liver, and tumor. Error bars represent mean ± SD.

To further explore the targeting characteristics of Glc-Alb, comparison studies using *in vivo* imaging and spatial transcriptomics between Man-Alb and Glc-Alb were conducted. First, to assess the cancer-targeting efficacy of Glc-Alb in the 4T1-bearing mouse model, we conducted a PET image-based evaluation. Moreover, inspired by previous studies suggesting that GLUT specificity for the carbon site might vary when glucose is introduced into nanoparticles, we designed comparative experiments. For this experiment, we constructed Glc-Alb using glucose, with azide introduced at the 2^nd^ (Glc(2)-Alb) and 6^th^ (Glc(6)-Alb) carbons 34,35. Experiments were also performed for comparative analysis with results obtained using the same 4T1 model as Man-Alb, as previously reported by our group 17. A previous study aimed to confirm uptake in the lung region due to metastasis rather than primary tumors. However, this study revealed uptake in the periphery of primary tumors by TAM (CD206 positive). Thus, through comparative analysis with Glc-Alb, we anticipated its ability to specifically discern the various environments associated with cancer. Evaluation of blood distribution trends for Alb and Glc(2)-Alb using PET imaging revealed similar patterns over time (**Figure 5A**). The minimal difference in distribution between albumin and Glc(2)-Alb suggests reduced GLUT selectivity attributed to glucose at the 2^nd^ position (**Figure 5D and Table S22, Blood**). Particularly, the uptake in the liver, which has a high GLUT expression, was lower than that of albumin (**Figure 5D and Table S22, Liver**). However, Man-Alb and Glc(6)-Alb showed contrasting results. An early distribution half-life, indicative of rapid blood elimination, was prominently observed for Man-Alb, aligning well with the initial high liver uptake (**Figure 5D, Blood and Liver**). Furthermore, in terms of cancer uptake, Glc(6)-Alb demonstrated an increasing pattern over time, whereas Man-Alb showed saturation (**Figure 5D and Table S22, Tumor**). An intriguing imaging observation was that for Man-Alb, the peripheral uptake in the tumor model appeared as a donut-shaped pattern in the transaxial imaging (**Figure 5A, Trans**). In contrast, Glc(6)-Alb showed an increased uptake by the inner tumor tissue on transaxial imaging. At 8 h, Glc(6)-Alb exhibited high blood retention, which decreased over time, indicating an increased uptake in the tumor. In the final 24-hour imaging, *ex vivo* biodistribution confirmed that Glc(6)-Alb exhibited over twice the uptake in the tumor compared to Man-Alb (Glc(6)-Alb = 4.84 ± 0.67 %ID/g, Man-Alb = 2.38 ± 0.3 %ID/g), with evidence of intestinal excretion (**Figure 5B and Table S23**). In contrast, Man-Alb exhibited the highest residual uptake in the liver. Based on the macroscopic patterns observed in the PET images, the overall uptake of Glc(6)-Alb by cancer tissue was considered to be indicative of cancer cells or immune cells overexpressing GLUT1. This result aligns with a previous study, which found that a glucose molecule attached via the C6 position onto a nanoparticle could bind to GLUT1, whereas attachment at other positions could not 34. In addition, considering the correlation demonstrated in a previous study between Man-Alb and CD206, we hypothesized that the uptake image around the cancer tissue would consist of anti-inflammatory immune cells (**Figure 5C**). Although nuclear medicine imaging provides high quantifiability, there are limitations to making speculations at the cellular or more microscale level using macroscale images. Therefore, similar to the preceding data, tissue evaluation was attempted using ST analysis, in which Glc(6)-Alb and Man-Alb were labeled with different fluorescences, co-injected, and subsequently sectioned for further analysis. Unless otherwise specified, Glc-Alb refers to Glc(6)-Alb.

### Spatial Clustering Analysis Reveals Distinct Uptake Patterns of Man-Alb and Glc-Alb

**Figure 6:**
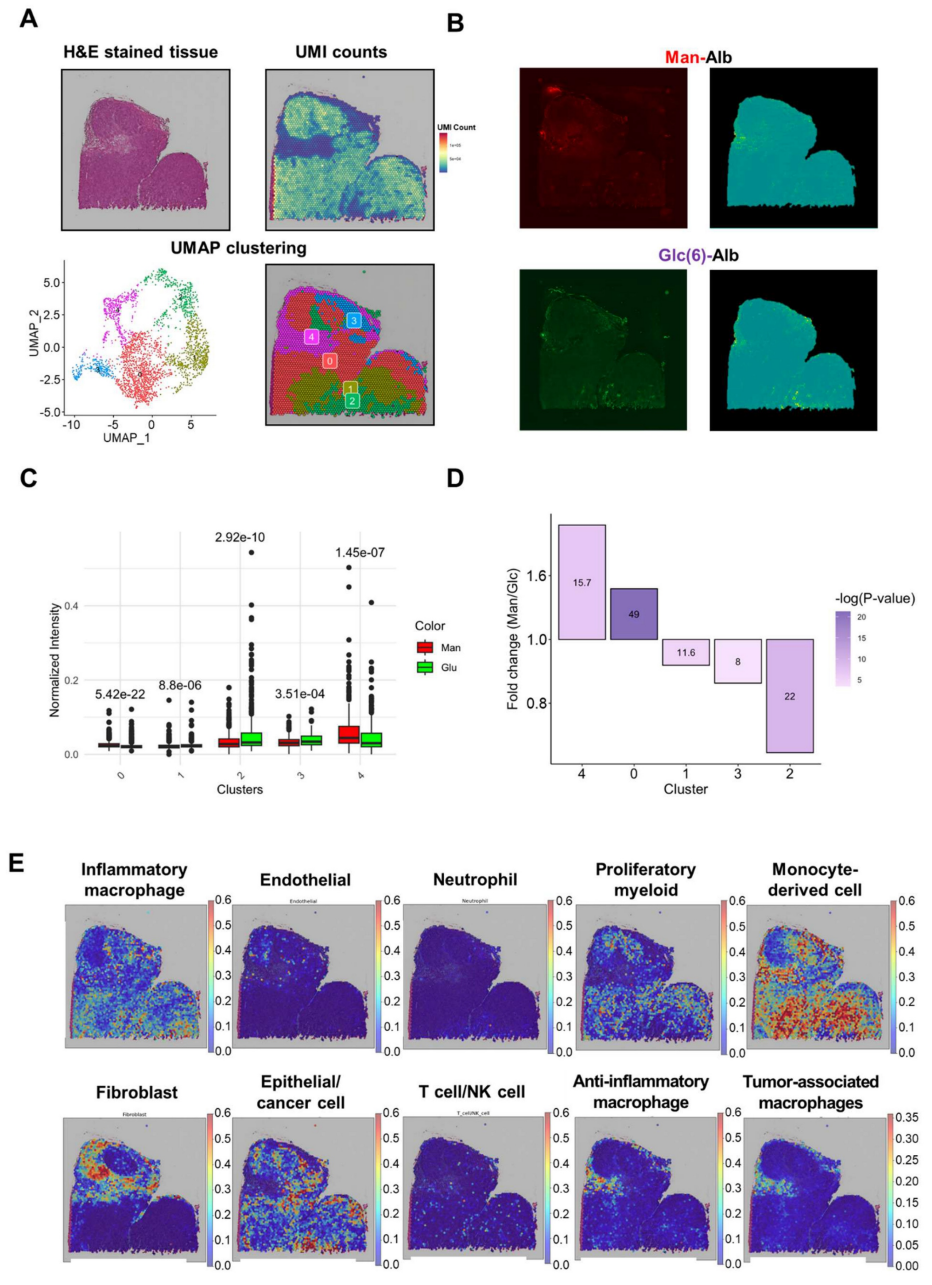
** Summary of ST library of Man+Glc sample.** (A) The images were derived from spatial transcriptomic (ST) data of the Man+Glc sample. Each image represents the H&E image (upper left), the UMI counts provided by SpaceRanger (upper left), and the spatial clustering analysis of the Man+Glc sample (below). UMAP projections of spatial clusters after data integration with the Man+Glc sample using the Seurat pipeline (left) and the distribution of spatial clusters according to sample (right). (B) Fluorescence images of the Man+Glc sample. Flamma fluor 648-conjugated mannose albumin and Flamma Fluor 488-conjugated glucose albumin nanoparticles are scanned with appropriate ex/em filters (left). Additionally, they mapped with ST spots using the SPADE algorithm (right). White arrows indicate unreliable FL signals, but yellow arrows indicate seemingly biologically meaningful FL signals. (C) Relative fluorescence signals of each cluster mapped by SPADE algorithm to ST libraries according to albumin nanoplatform. Error bars represent mean ± SD. (D) Mean intensities of albumin fluorescence signals in each cluster. P-values between mannose and glucose albumin fluorescence signals. (E) CellDART results for the Man+Glc sample. The original 4T1 scRNA-seq reference for CellDART execution contained only nine cell types, among which the original 'Anti-inflammatory_macrophages' was divided into the newly defined 'Anti-inflammatory_macrophages' and 'Tumor-associated_macrophages' for non-TAM-like cells and TAM-like cells, respectively.

The same ST study was performed using different types of fluorescently labeled Man-Alb and Glc-Alb co-injected into 4T1 tumor-bearing mice. When mapping FL intensities to ST spots using the SPADE algorithm, Man-Alb uptake was slightly higher in the upper left region of the tumor section, which was clustered at 4. Glc-Alb uptake was more distinct in the lower-middle region of the tumor section, which was clustered as 2 (**Figure 6**).

### Integrative Analysis of Clusters and FL Signals Reveals Distinct Uptake Patterns Between Man-Alb and Glc-Alb

When comparing the fluorescence intensities of Man-Alb and Glc(6)-Alb within each cluster, significant differences in albumin uptake were observed across all clusters. Specifically, Man-Alb uptake was notably higher in clusters 0 and 4, whereas Glc(6)-Alb uptake was more pronounced in clusters 1, 2, and 3 (**Figure 6A, B**). Notably, Man-Alb uptake in cluster 4 was 1.358-fold higher than that of Glc(6)-Alb, whereas it was 0.645-fold lower in cluster 2 compared to Glc(6)-Alb (**Figure 6C, D**).

### Specific TME Cell Types Associated with Man-Alb and Glc-Alb

The CellDART results demonstrated a distribution similar to that of Man-Alb and Gal-Alb co-injected tumor samples (Man+Gal). When exploring the fluorescence pattern and CellDART results, a high Man-Alb uptake region (cluster 4) demonstrated an intensive distribution of anti-inflammatory and TAMs. The region with high Glc(6)-Alb uptake showed epithelial and cancer cell distribution (**Figure 6E, Figure S10**). Similarly, in the correlation analysis between the cell types in the TME estimated by CellDART and fluorescence signals, anti-inflammatory macrophages and TAMs exhibited a modest positive correlation with Man-Alb uptake in the Man+Gal sample (**Table S24**). Furthermore, when we conducted the same analysis within each cluster, cluster 4 yielded similar findings (**Tables S25-29**).

**Figure 7:**
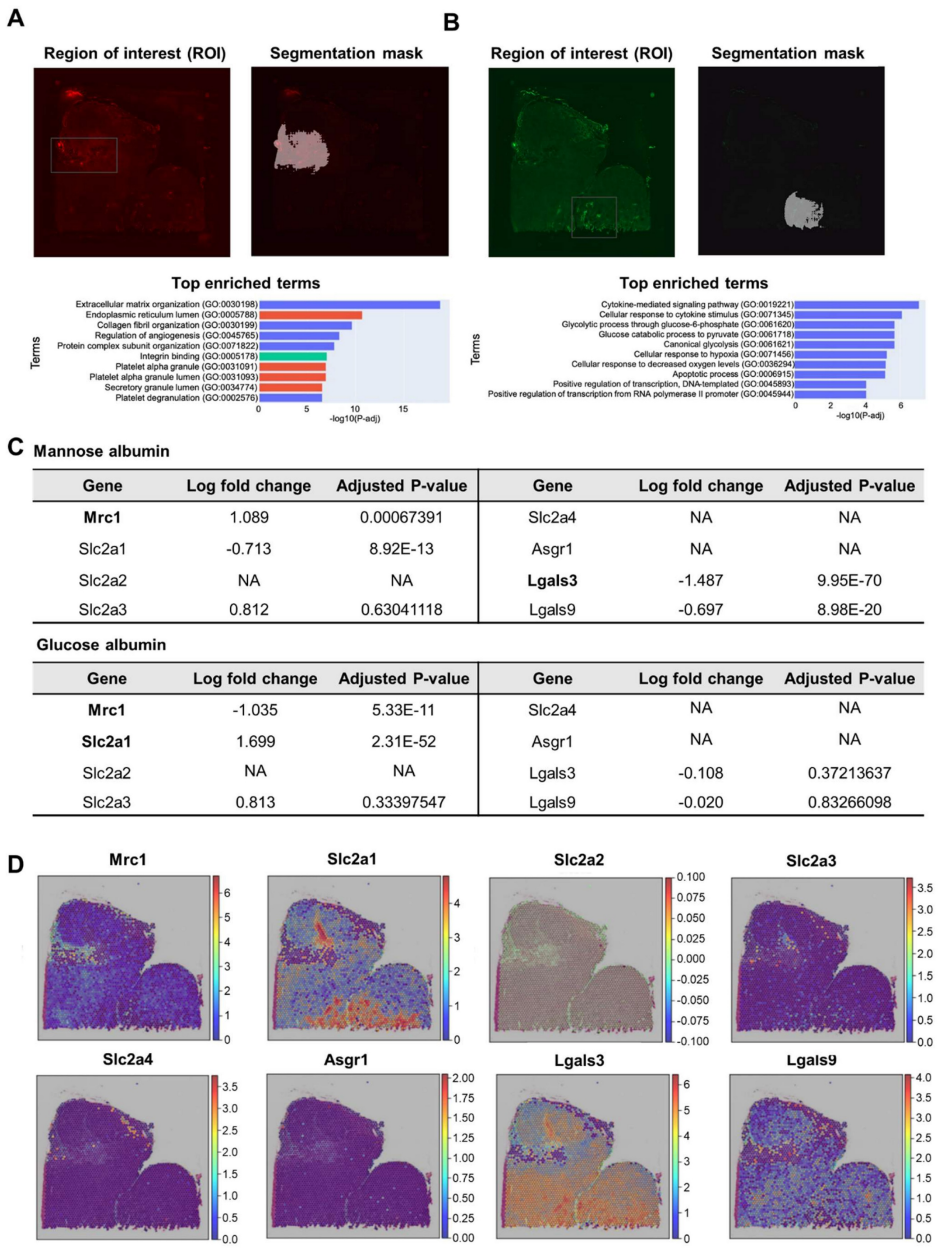
** Analysis of high CAN-DGIT uptake regions of the Man+Glc sample.** (A) IAMSAM analysis of relatively high mannose albumin uptake region. The characteristic uptake region of mannose albumin was pointed as a gray rectangular box (upper left). When this region is applied in a segment-anything model, distinct patterns of albumin uptake in the region are segmented (upper right). Differentially expressed genes in this ROI were listed and used for gene set enrichment analysis (below). (B) The same analysis was performed in the glucose albumin distribution image. (C) Table of log fold change and adjusted p-values for comparing gene expression levels of each glycan-related gene between segmented ROI from IAMSAM and the rest region (D) Expressions of mannose receptor, glucose transporter, and asialoglycoprotein receptor-related genes.

In the IAMSAM analysis of the distinct uptake portion of Man-Alb and Glc(6)-Alb, similar GO terms were identified, as in the previous Man+Gal sample (**Figure 7A, B, Table S30, 31**). In the list of differentially expressed genes in each ROI, Mrc1 was upregulated, and Lgals3 was downregulated in the Man-Alb distinct region. In the distinct Glc(6)-Alb region, Slc2a1 was upregulated and Mrc1 was downregulated (**Figure 7C**). When comparing the expression patterns between the expression relevant to each receptor and the FL intensity, the uptake of Man-Alb or Glc(6)-Alb was similar to the expression of each receptor gene (**Figure 7D**). When the same analysis was applied to genes encoding glycan-binding proteins, regions with high mannose albumin intake showed similar results to the previous one, with notable Mrc2 and Clec2d overexpression. Conversely, in areas with predominant Glc-Alb intake, Lgals3 overexpression was prominent (**Tables S32, S23, Figure S11**).

### Enhanced Target Efficiency Using CAN-DGIT Approach

Building on the results demonstrating the selective distribution of glycosylated albumins in tumor-associated macrophages (TAMs), we next examined whether these nanoparticles could improve the therapeutic efficacy of a macrophage-depleting agent within the tumor.

**Figure 8:**
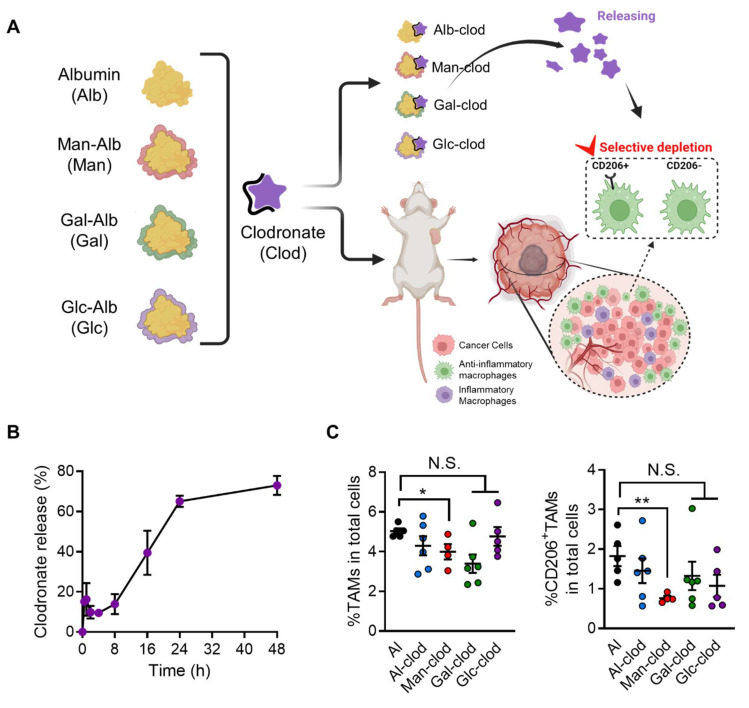
**Evaluation of the efficacy of the CAN-DGIT approach based on ST analysis in an animal model.** (A) Each glycosylated albumin complexed with clodronate, as configured in Figure 1A, to form respective complexes. Drug incorporation and release were assessed through release tests. Additionally, each complex was administered to 4T1-bearing mice to evaluate specific cell targeting in tumor tissues confirmed through ST analysis. (B) Releasing profile of the Man-Alb/clodronate drug complex. (C) Percentages of TAMs (CD45^+^CD11b^+^Ly6g^-^F4/80^+^; left) and CD206^+^TAMs (right) in 4T1-bearing mice treated with either Alb/clodronate complex (n = 6 mice), Man-Alb/clodronate complex (n = 4 mice), Gal-Alb/clodronate complex (n = 6 mice), Glc-Alb/clodronate complex (n = 5 mice) or albumin control (n = 5 mice). All data represented as mean ± S.E.M. Statistical significance was determined by two-tailed t-tests. *P < 0.05, **P < 0.01. N.S., nonsignificant.

Specifically, we evaluated the clodronate-loading capabilities of various glycosylated albumins—known to effectively target and deplete macrophages—in 4T1 tumor-bearing mice following intravenous injection. As depicted in Figure 8A, each glycosylated albumin was complexed with clodronate to form a stable nanoparticle-drug formulation, and their release profiles were analyzed (**Figure 8B, Figure S12**). For clarity, the clodronate release profiles of Gal-Alb and Glc-Alb were also evaluated, and the results together with the Man-Alb/Clodronate data are provided in Supplementary Figure S13. Notably, these profiles did not differ significantly among the different glycation patterns, suggesting that surface carbohydrate composition did not adversely affect drug release kinetics. After confirming stability, we administered each complex three times at three-day intervals to the tumor-bearing mice. Strikingly, the Man-Alb/clodronate complex (Man-clod) induced a marked reduction in tumor-infiltrating TAMs compared to the other glycosylated albumin/clodronate formulations (**Figure 8C**). In addition, Man-clod treatment effectively depleted CD206+ TAMs—those often associated with pro-tumoral, anti-inflammatory phenotypes—underscoring the specificity and therapeutic relevance of mannose-receptor targeting in the tumor microenvironment.

By demonstrating that fine-tuned glycosylation on an albumin nanoplatform can enhance the localized depletion of TAMs, these results highlight the promise of the CAN-DGIT strategy for precise immunomodulation. Furthermore, our data suggest that coupling a targeted nanoplatform with clodronate or other immunoregulatory agents may be a powerful approach to reprogramming the TME toward improved anticancer efficacy.

## Discussion

In recent years, a variety of methodological advances and structural modifications have been implemented to optimize nanoparticle-based strategies for targeting the TME 29-32. In this study, we synthesized and characterized an albumin nanoplatform engineered by the distinct glycosylation of different monosaccharides (mannose, galactose, and glucose) to enhance selective cell targeting in the TME. The ability to precisely control the number and type of glycosylations on albumin nanoplatforms is crucial for enhancing their specific targeting and circulation properties. Previous studies have employed electrostatic interactions, direct amide linkages using amino group-conjugated monosaccharides, and thiourea linkages, which complicate the precise modulation of the number of attached monosaccharides. Mannose glycosylation with chitosan-functionalized nanoparticles using electrostatic interactions makes it difficult to control the number of mannose molecules attached to the nanoparticles 36.

Dhanikula et al. synthesized a glycosylated dendrimer by conjugating d-glycosamine to a hydroxyl group on the dendrimer. The authors used the same molar ratio of dendrimer and D-glucose; however, because there are multiple active sites on the dendrimers, it is difficult to ensure that one dendrimer has one glucose molecule on the nanoparticle 37. Liu et al. later reported glycosylated iron oxide nanoparticles with various monosaccharide moieties, but their method also faced difficulties in determining the exact number of monosaccharides per nanoparticle and the precise molar ratios 38. However, this method makes it difficult to determine the exact number of monosaccharides on each nanoparticle and accurately determine the molar ratio between the NPs and monosaccharides. Moreover, precise control of the targeting moiety is important because a high density of target molecules does not necessarily lead to significant accumulation at the desired location, such as the tumor site 39-41**.** Our click chemistry-based approach allowed us to introduce a predefined number of carbohydrates onto the albumin surface, ranging from 1 to 6, without affecting the size or shape of the nanoparticles (**Figure S14**). This enabled us to investigate the effects of different glycosylation patterns with the matching number of monosaccharides on one albumin on the biodistribution, cellular uptake, and gene expression of the albumin nanoplatforms *in vivo.*

Similar to previous studies, we aimed to determine the numbers of mannose, galactose, and glucose based on precisely set glycosylation numbers determined by the number of ADIBO, utilizing nuclear medicine imaging techniques. We employed an albumin-based nanoplatform with 11 ADIBO, along with six glycosylation targets, as used in previous experiments. As shown in Figure 1, similar to earlier papers, AD^11^-Albumin exhibited elevated residual amounts in the liver at 24 h, whereas Man^6^-Alb demonstrated imaging consistent with previous studies. Similarly, Gal^6^-Alb and Glc^6^-Alb exhibited distinct *in vivo* distribution patterns (**Figure S15**). One of our aims was to emphasize the changes in distribution solely due to surface glycosylation while maintaining the properties of the control group, albumin. Hence, we used a clickable albumin platform with 6-7 ADIBO groups and introduced four different carbohydrates to produce glycosylated albumin. Experimentally, we confirmed that for *in vivo* cell targeting, albumin with six ADIBO should be used as a reference, minimizing non-specific liver cell uptake by the RES. Therefore, all albumin nanoplatforms in this study maintained a consistent configuration of six ADIBO with four glycosylation targets to emphasize the changes in distribution due to surface glycosylation, while preserving the properties of albumin. Our nanoplatform was shown to have precise control over the number of glycosylations per nanoparticle, enabling the development of an *in vivo* cell-specific target.

In this study, by combining PET for whole-body kinetics, fluorescence for microscopic localization, and spatial transcriptomics for molecular correlation, we established a top-down imaging framework. This integrative strategy facilitated comprehensive evaluation of glycosylated albumins within the tumor microenvironment. Firstly, to confirm the different biodistributions and targeting abilities of various glycosylated albumins, quantitative PET *in vivo* imaging was performed. The results showed that Man-Alb had the highest liver uptake due to CD206 expression in liver Kupffer cells. Initially, Gal-Alb also had high liver uptake but was soon excreted into the GI tract. Glc-Alb showed the highest targeting ability in tumor tissues. Most previous studies on glycosylated nanoparticles have thoroughly explored the *in vivo* biodistribution of nanoparticles rather than focusing on target-tissue targeting. Liu et al. demonstrated higher glucosylated IONP uptake in tumor tissues by enhanced T2 signals on magnetic resonance imaging 38. However, the study did not explore the different biodistributions of various glycosylated nanoparticles, which is crucial for evaluating the utility of drug delivery carriers. Frigell et al. reported the PET imaging of glucosylated gold nanoparticles by radiolabeling with ^68^Ga. They compared different types of blood-brain barrier-permeable neuropeptide conjugates and showed differences in their biodistribution and brain-targeting efficiency. This study focused on the effects of neuropeptide conjugation rather than glycosylation 42. In our study, the only difference among Glc-Alb, Gal-Alb, and Man-Alb was the type of monosaccharide used. Based on *in vivo* distribution studies using PET imaging, we evaluated how the distinct glycosylation employed in this study behaves within the body. This analysis aligns with the recent trends in organ-targeting nanoparticles. In recent years, organ-specific delivery of nanoparticles has been developed. For example, the selective organ-targeting (SORT) system is a lipid nanoparticle system designed to target the liver, spleen, and lungs using different types of lipid components. However, this method does not utilize targeting moieties to specifically target cells, and the mechanisms for targeting these cells have not been well elucidated 43.

In this study, we utilized ST techniques to analyze the cell types and genes associated with the tissue distribution of various glycosylated albumins. To investigate the DEGs and cell types present in locations with high glycosylated albumin concentrations, we utilized a previously established ST data analysis method. This analysis was conducted on cancer tissue samples simultaneously administered with mannose, galactose albumin, or glucose albumin. Results revealed that by aligning the fluorescence image with the spatial transcript spots, partial correction of false signals and noise in the fluorescence image was possible. Furthermore, we confirmed that the distribution patterns of these two types of glycosylated albumins varied within each tissue sample. To better understand the characteristics of regions with distinct distributions of glycosylated albumin, we utilized UMAP clustering to divide the tissue space into five clusters that exhibit similar biological characteristics. Clustering allowed us to identify areas where each glycosylated albumin was predominantly distributed and gain insights into the unique characteristics of each cluster by analyzing the DEGs specific to each cluster. Moreover, when we compared the distribution of each glycosylated albumin with the identified clusters, we observed that mannose albumin was primarily concentrated in cluster 4. In contrast, galactose and glucose albumin were predominantly found in cluster 2. Using CellDART and IAMSAM analyses, we explored the transcripts and cell types associated with glycosylated albumin uptake. Notably, mannose albumin exhibited a distribution pattern similar to that of anti-inflammatory macrophages and TAMs, predominantly in regions rich in extracellular matrix-related genes. In contrast, galactose and glucose albumin displayed distribution patterns akin to those of cancer/epithelial cells. We confirmed that genes related to various metabolic processes associated with cancer were concentrated in these areas. Finally, our investigation into the expression patterns of different receptors and the distribution of glycosylated albumin revealed that mannose albumin distribution was correlated with Mrc1, whereas the galactose and glucose albumin distribution was associated with Slc2a1. These spatial transcriptome analysis findings demonstrate the potential to selectively target different cell types within tumors by leveraging the glycosylation of albumin nanoparticles. To further examine potential differences between galactose- and glucose-conjugated albumins, we integrated the ST datasets of Man+Gal and Man+Glc samples using the scVI algorithm. Cross-dataset transfer of Gal and Glc scores demonstrated that the reconstructed Glc-Alb and Gal-Alb signals closely resembled the corresponding native scores and were exclusively localized within cancer epithelial cells (**Figure S16**). This suggests that both Gal-Alb and Glc-Alb converge toward epithelial cell targeting within the tumor microenvironment.

Based on these results, the most evident distinction between Man-Alb and Glc-Alb was their potential for targeting macrophage subtypes. Consequently, when evaluating the targeting efficiency at the cellular level in M0, M1, and M2, the differences were confirmed by *in vitro* fluorescence experiments. The selective targeting of M1 by Glc-Alb and Man-Alb was identified (**Figure S17**). When further validating the differences under M0 and M1 polarization conditions, Glc-Alb demonstrated over 10-fold higher targeting efficiency in M1 compared to albumin without glucose (**Figure S18A, B**). Additionally, the M2 targeting ability of CD206, as identified in previous studies, was validated using this platform, showing a complete match with the IHC results of cancer tissue and fluorescently labeled Man-Alb in adjacent tissue sections (**Figure S19**). When the distributions of CD206 and Man-Alb in six different tumors were compared in the same section using immunofluorescence, it was confirmed that the uptake was higher in areas where CD206 was highly expressed (**Figure S20**). The significance of this approach lies in its ability to predict the targeting efficacy within a tissue through ST analysis without the need for basic experiments for target assessment or mechanistic evaluation. In other words, this approach may provide a direct analytical method for correlating cellular-level mechanistic evaluations with efficacy results in animal models.

We were able to effectively compare the targeting characteristics of glycosylated albumins using our developed analytical platforms, such as SPADE, CellDART, and IAMSAM. We must acknowledge that our comparison was based on a single sample. To ensure reproducibility of our experiment, it is essential to increase the sample size and conduct a thorough analysis to validate our results. Even with this limited comparison, the distinct similarity in the distribution patterns of Man-Alb and other glycosylated albumins indicates that our developed method effectively explains the distribution of nanoparticles to a certain extent. Complementary to this, emerging work has expanded albumin nanoplatform applications toward transformable nanocapsules and immune conjugates, which further underscores the versatility of this biomaterial 44-46. Integrating these advances with our present glycosylation-based approach strengthens the rationale for albumin as a modular nanoplatform that can be rationally engineered to interrogate and therapeutically exploit the tumor microenvironment. Recently, a proof-of-concept study was conducted combining various glycans on the surface using glycocalyx-mimicking GlyNPs as a platform, enabling the screening and identification of glyconanoparticles targeting various types of cancer 47**.** However, to demonstrate this experimentally, extensive *in vitro* screening and *in vivo* efficacy evaluation using fluorescence imaging are required. As mentioned earlier, utilizing the research nanoplatform and analytical methods presented in this study allows for a rapid assessment of distribution differences at the cellular level, with minimal samples for distinct glycosylation.

## Conclusion

Our methods, CAN-DGIT, and spatial transcriptomics-based evaluation are ideal for optimizing delivery systems with complex targeting properties, such as glycosylated nanoparticles, and for identifying specific application sites for these systems. Furthermore, we believe that spatial transcriptomics-based microscopic evaluation of drug delivery systems can further validate and support the findings of *in vivo* imaging. Using our method, further optimization is warranted for targeting specific TMEs, such as TAMs and cancer cells, by fine-tuning the degree of glycosylation and the combination of conjugated saccharides. We anticipate that our method will significantly contribute to the understanding of the TME and the development of actively targeted nanoparticles.

## Supplementary Material

Supplementary figures and tables.
