# Supplementary material for: Engineering and evaluation of precision-glycosylated clickable albumin nanoplatform for targeting the tumor microenvironment
Source: Theranostics. 2026 Jan 1;16(3):1482–503. doi: 10.7150/thno.123973 (PMC12679570; doi:10.7150/thno.123973)
Supplement: Supplementary file 1 — Supplementary figures and tables. [file thnov16p1482s1.pdf]

Supplementary Materials

1. Supplemental Figures

Structure of sugar-azide and molecular weight

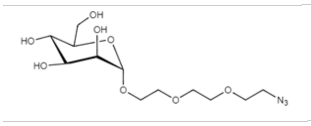

alpha-Man-TEG-N_3

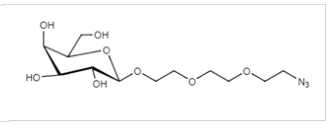

beta-Gal-TEG-N_3

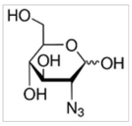

2-Azido-2-deoxy-D-glucose

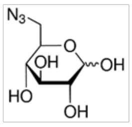

6-Azido-6-deoxy-D-glucose

	Mw (g/mol)
alpha-Man-TEG-N3	337.33
beta-Gal-TEG-N3	337.33
6(2)-Azido-6(2)-deoxy-D-glucose	205.17

Figure S1. Structure and molecular weight of sugar-azide.

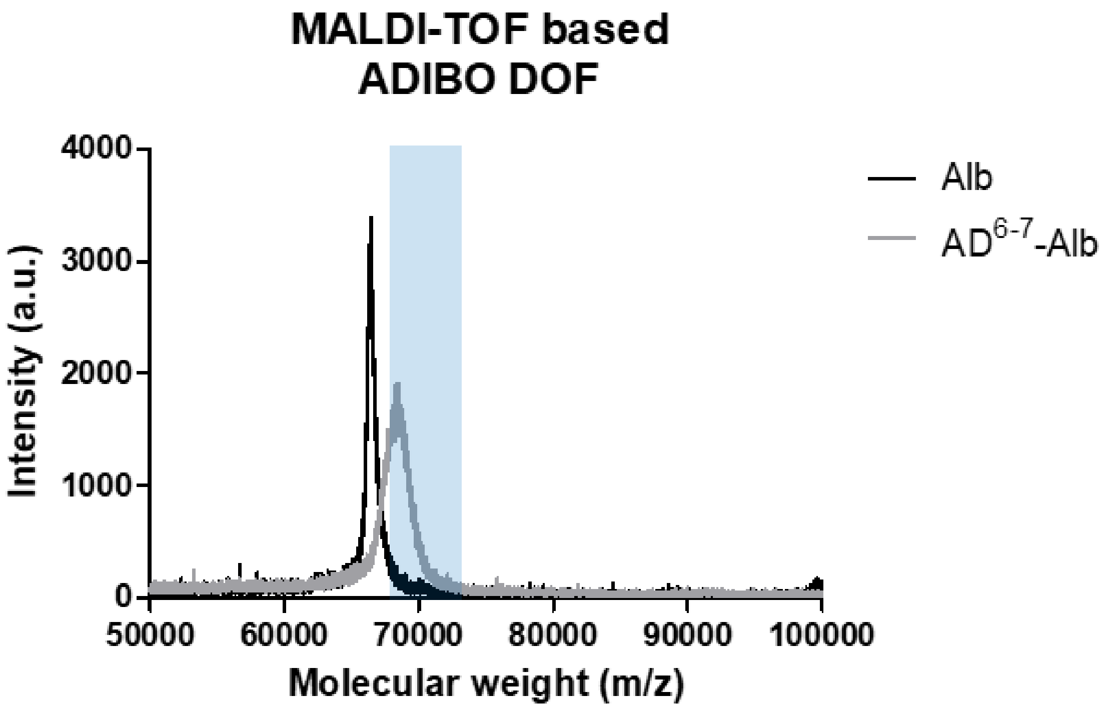

Figure S2. MALDI-TOF-based molecular weight measurement. In the line graph, the black line represents albumin, while the gray line corresponds to the ADIBO-modified albumin.

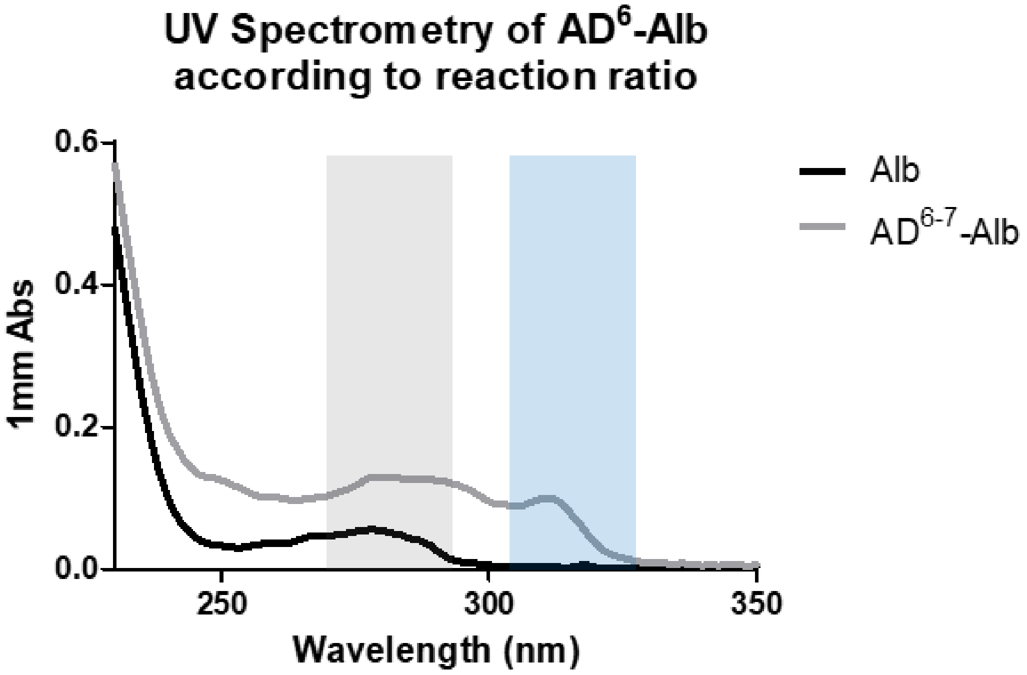

Figure S3. UV-based molecular weight measurement. In the line graph, the black line represents albumin, while the gray line corresponds to the ADIBO-modified albumin.

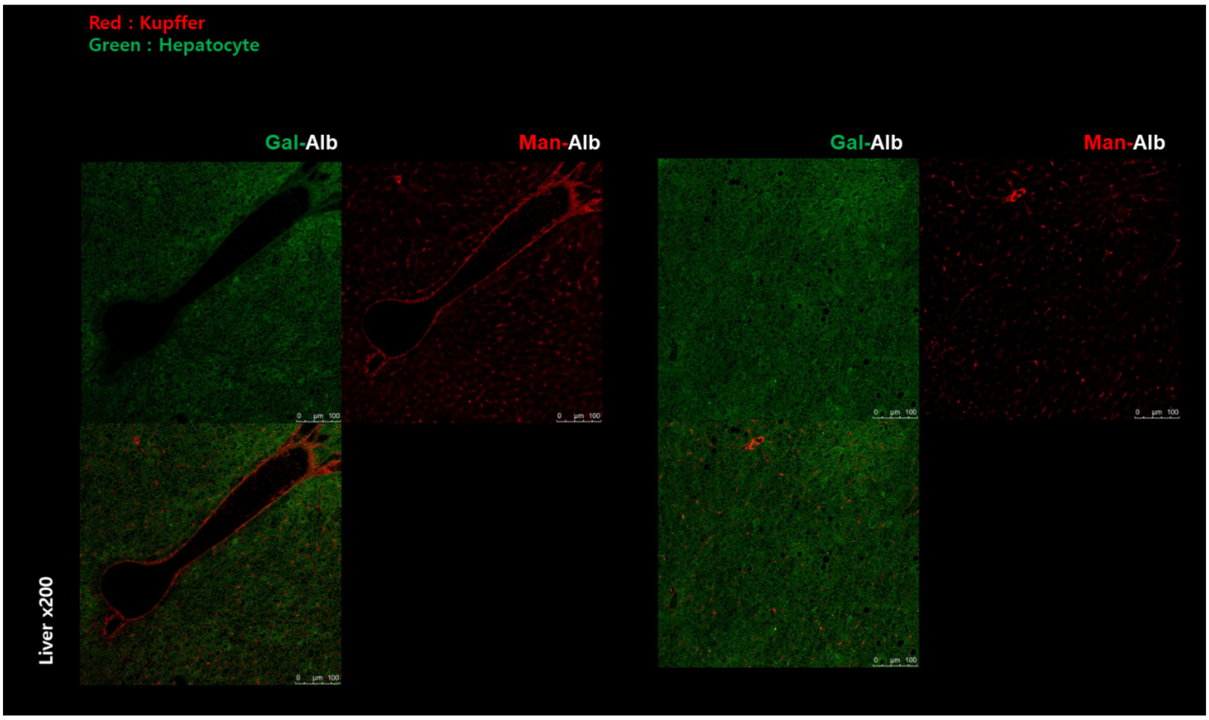

Figure S4. Fluorescence image at a 200× magnification for Figure 2B.

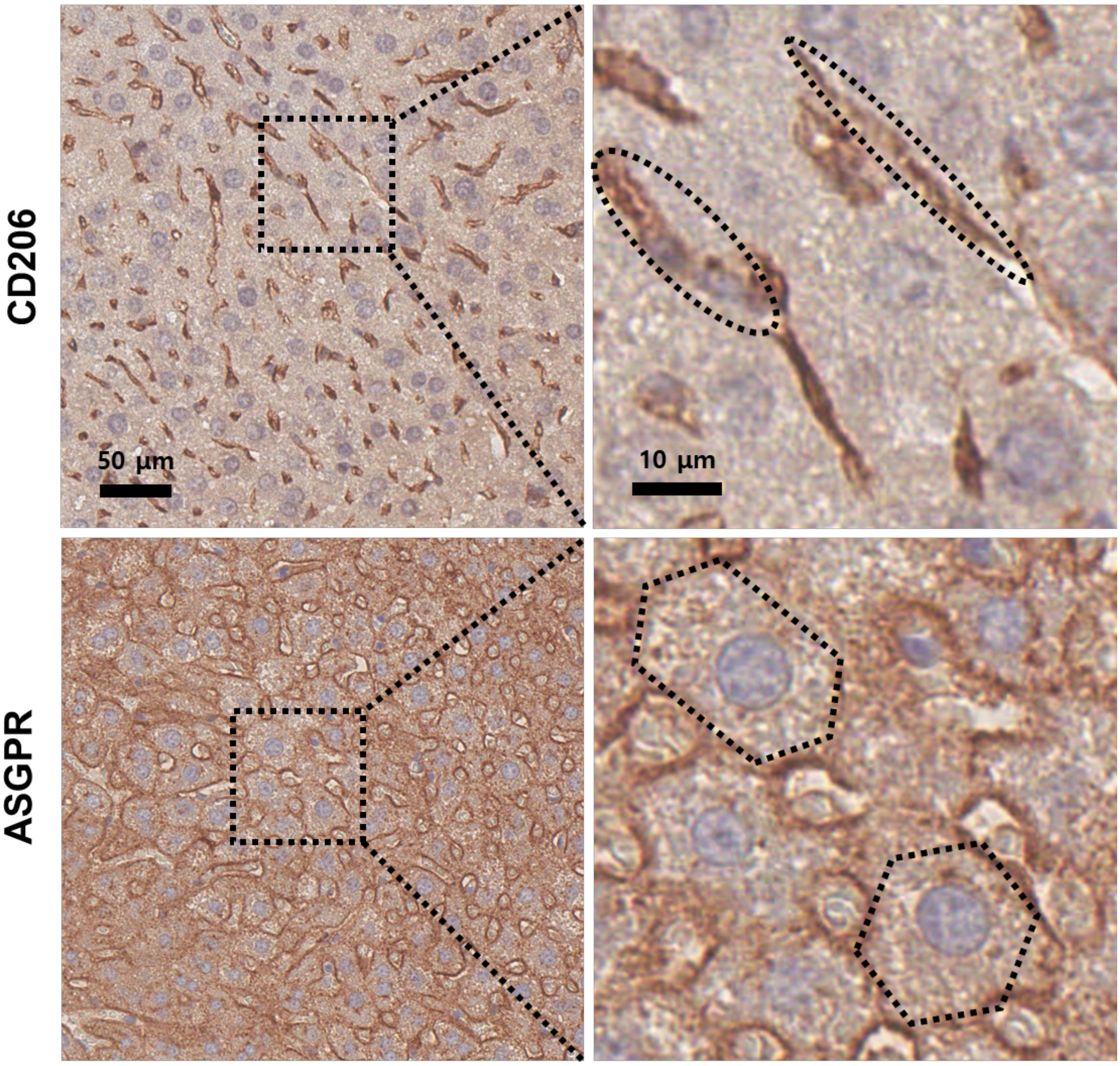

Figure S5. Immunohistochemistry images obtained by staining with anti-CD206 antibody and anti-ASGPR antibody. The dotted ellipses and pentagons respectively represent Kupffer cells and hepatocytes. Scale bars indicate 50 µm and 10 µm for the magnified images.

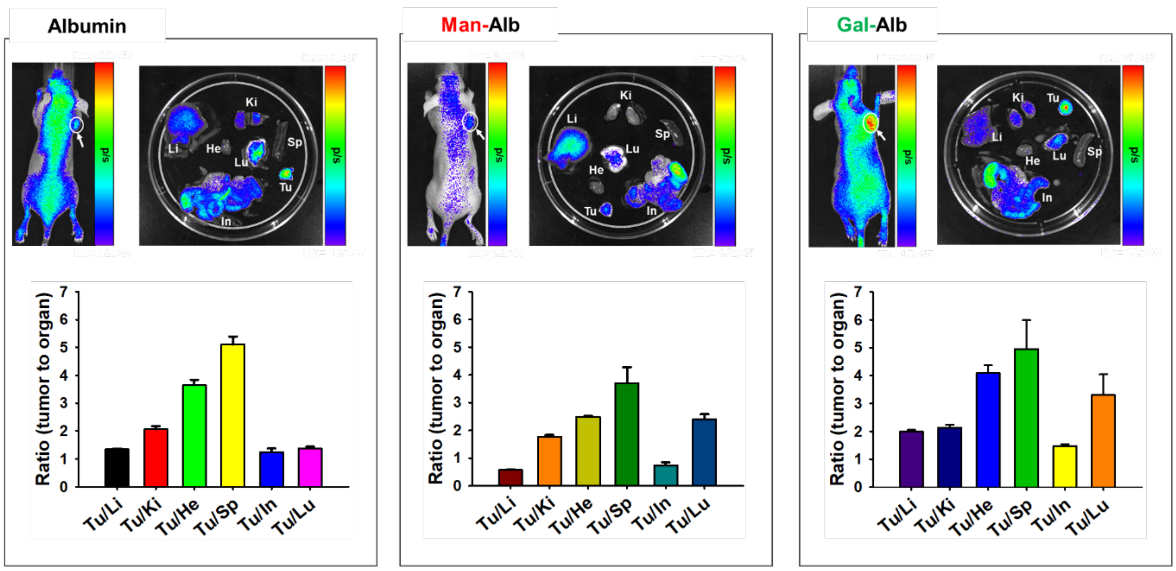

Figure S6. ***In vivo*** **and** ***ex vivo*** **fluorescence imaging** (Tu: Tumor, Li: Liver, Ki: Kidney, He: Heart, Sp: Spleen, In: Intestine, Lu: Lung).

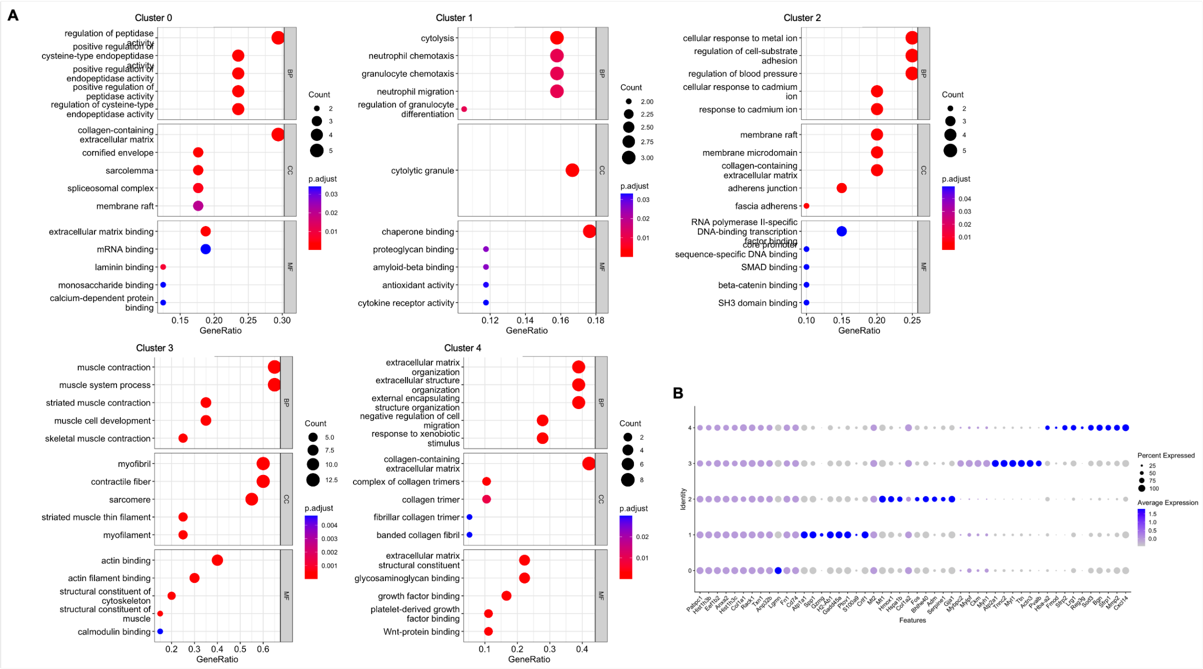

Figure S7. (A) GO analysis of each cluster and (B) dot plot of the top 10 DEGs of each cluster.

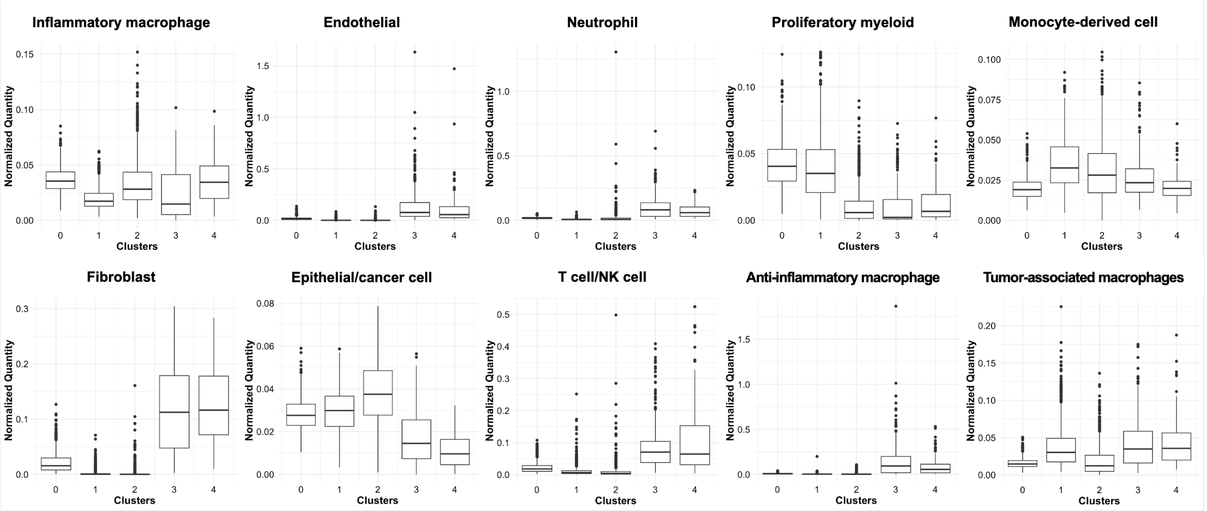

Figure S8. Boxplots of mean quantities of each cell type with CellDART analysis by clusters in the Man+Gal sample.

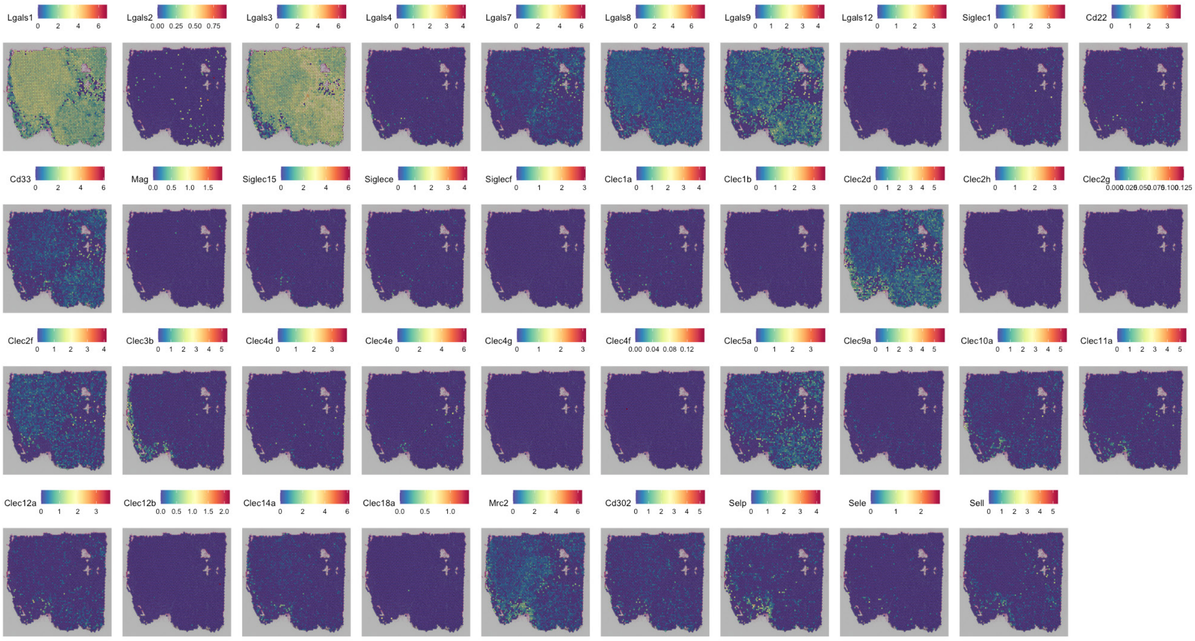

Figure S9. Expressions of glycan binding genes in the Man+Gal sample.

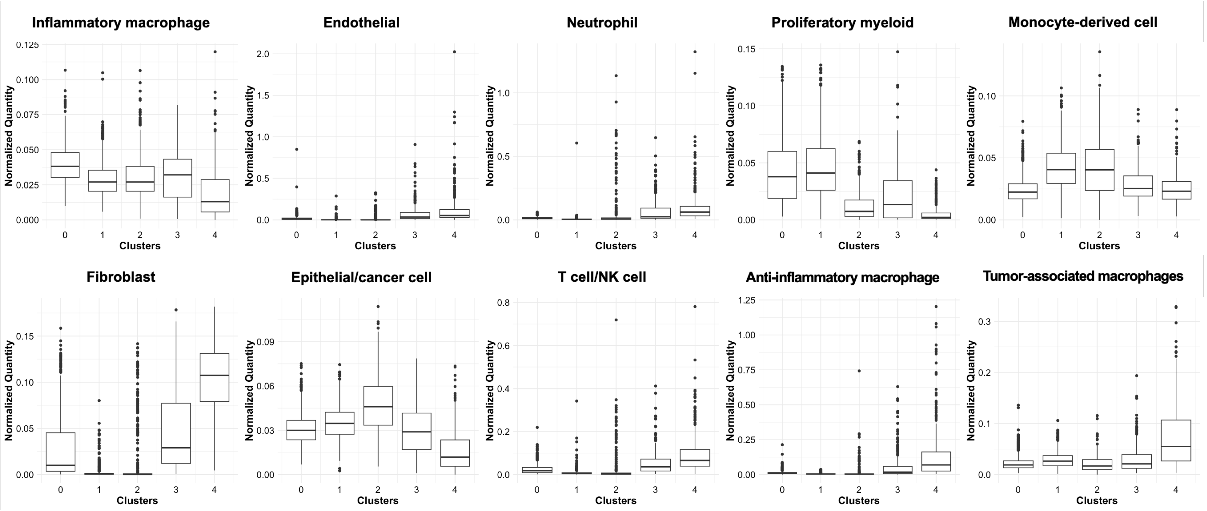

Figure S10. Boxplots of mean quantities of each cell type with CellDART analysis by clusters in the Man+Glc sample.

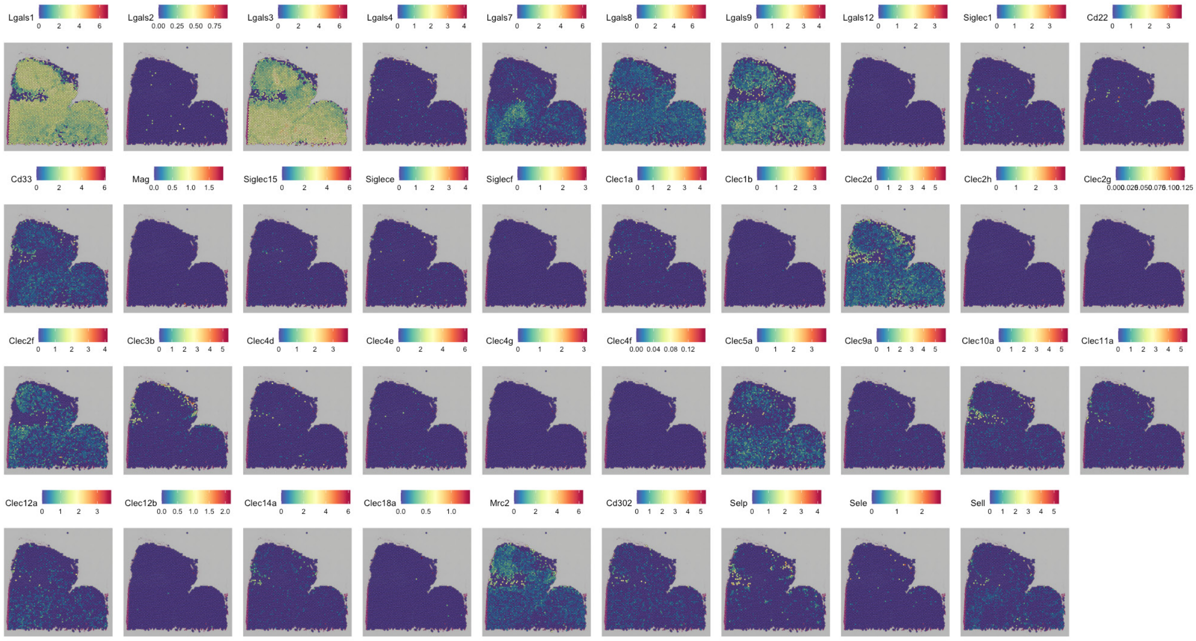

Figure S11. Expressions of glycan binding genes in the Man+Glc sample.

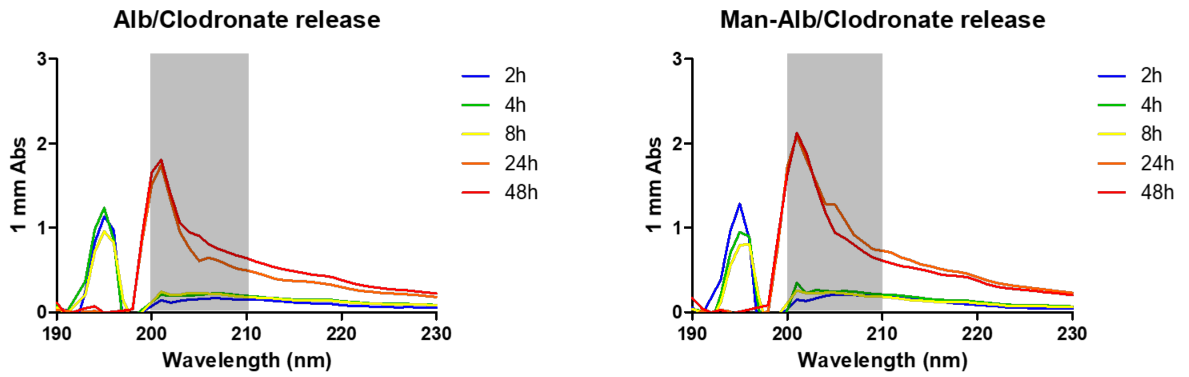

Figure S12. Clodronate-loaded complexes of albumin and Man-Alb were confirmed via UV spectroscopy. The release was evaluated by measuring UV absorbance at 205 nm. Samples were taken over time and purified, and the increase in absorbance at 205 nm in the filtrate obtained from the centrifugal filter was measured. Both Alb and Man-Alb exhibited similar releasing profiles.

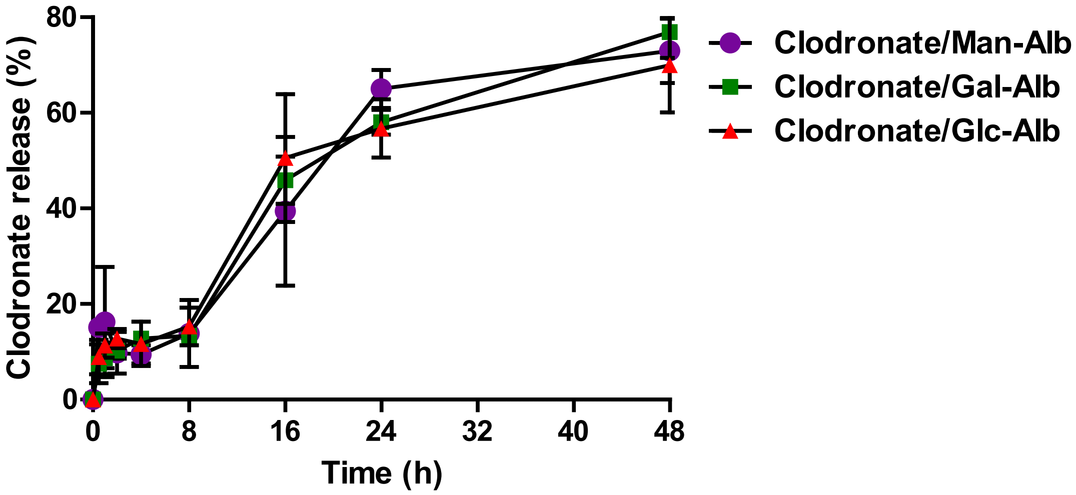

Figure S13. Clodronate release profiles from Man-Alb, Gal-Alb, and Glc-Alb nanoplatforms. All formulations exhibited similar sustained release kinetics, reaching ~70–80% release at 48 h. Data are presented as mean ± SD (n = 3).

Sample	Concentration(mg/ml)	Size(nm)
ADIBO-HSA		7.146±1.152
Glc-Alb	20	6.918±1.092
Gal-Alb		7.120±1.121
Man-Alb		7.013±1.108

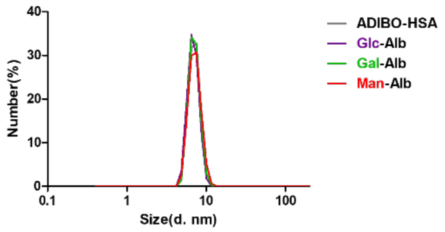

Sample	Zeta-potential(mV)
ADIBO-HSA	-19.3±6.28
Glc-Alb	-22.2±7.24
Gal-Alb	-21.8±9.14
Man-Alb	-21.3±8.48

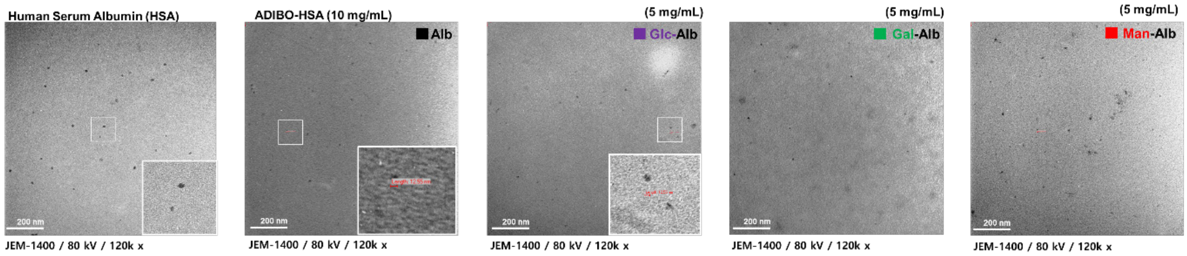

Figure S14. Morphological and physicochemical characterization of glycosylated albumins. TEM images of HSA, ADIBO-HSA, Glc-Alb, Man-Alb, and Gal-Alb confirmed their nanoparticle morphology at high resolution. DLS analysis revealed consistent hydrodynamic sizes across all glycosylated forms, and ζ-potential measurements showed no significant differences among Glc-, Man-, and Gal-modified albumins, indicating comparable surface charge characteristics.

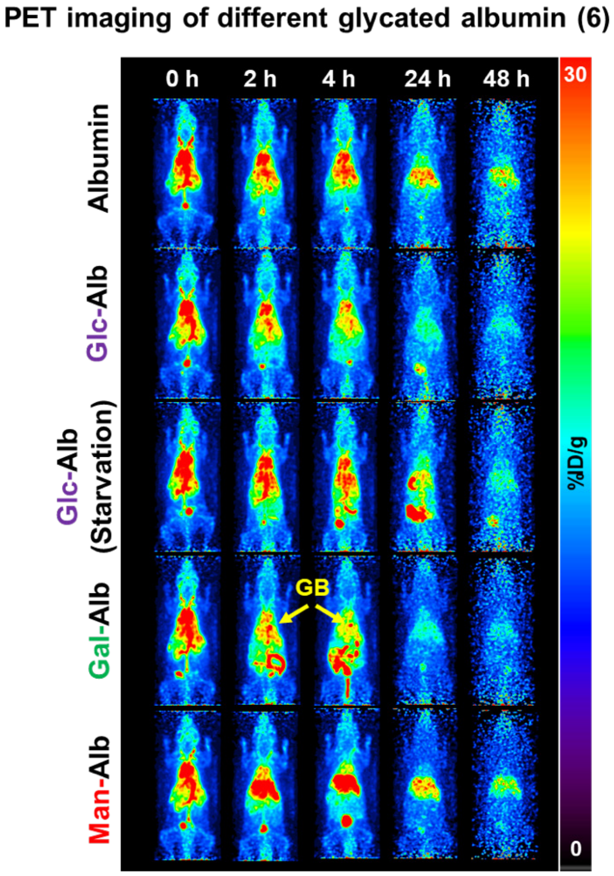

Figure S15. Representative PET images of ^{64}Cu-labeled distinct glycated albumin, featuring 11 ADIBO functional groups with 6 distinct glycation levels, in normal mice following intravenous administration at various time points (0, 2, 4, 24, and 48 h; n = 4 for each group). The yellow arrow indicates the gallbladder (GB)

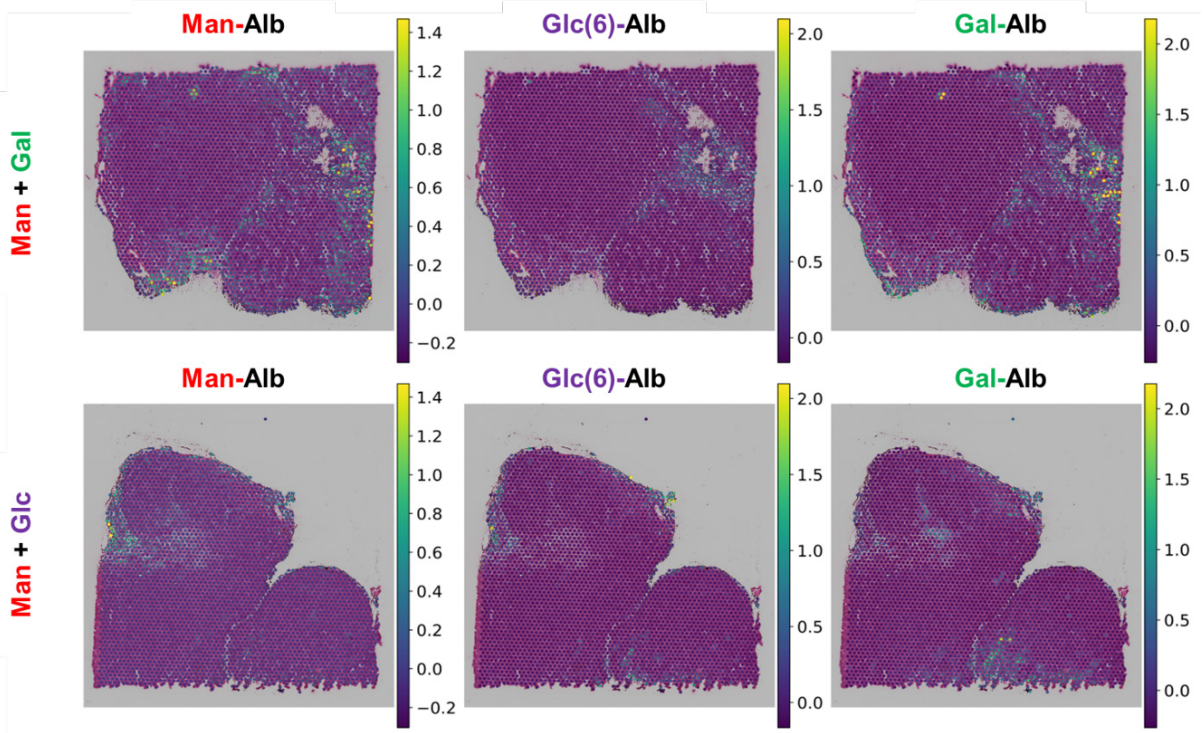

Figure S16. The distribution of each drug (Man-Alb, Glc-Alb, Gal-Alb) depending on the sample condition (Man+Gal, Man+Glc). After integration of two ST datasets using scVI, we transferred Gal scores from Man+Gal to Man+Glc and Glc scores vice versa. Each target spot was assigned the mean score of its five nearest reference spots in the scVI embedding space.

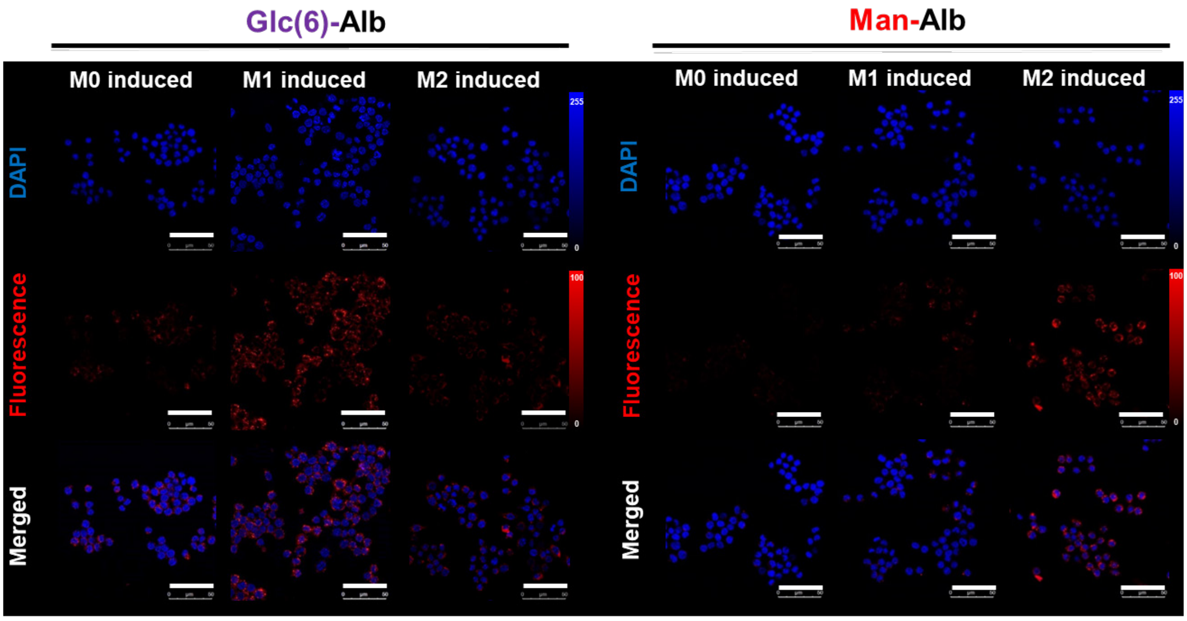

Figure S17. Differential targeting of macrophage polarization using Glc-Alb and Man-Alb. Glc-Alb demonstrated the highest uptake in M1-induced polarized macrophages, while Man-Alb demonstrated the highest uptake in M2-induced polarized macrophages. It was confirmed that each state had high selectivity in uptake by different glycation-modified albumins. Scale bar = 50 μm.

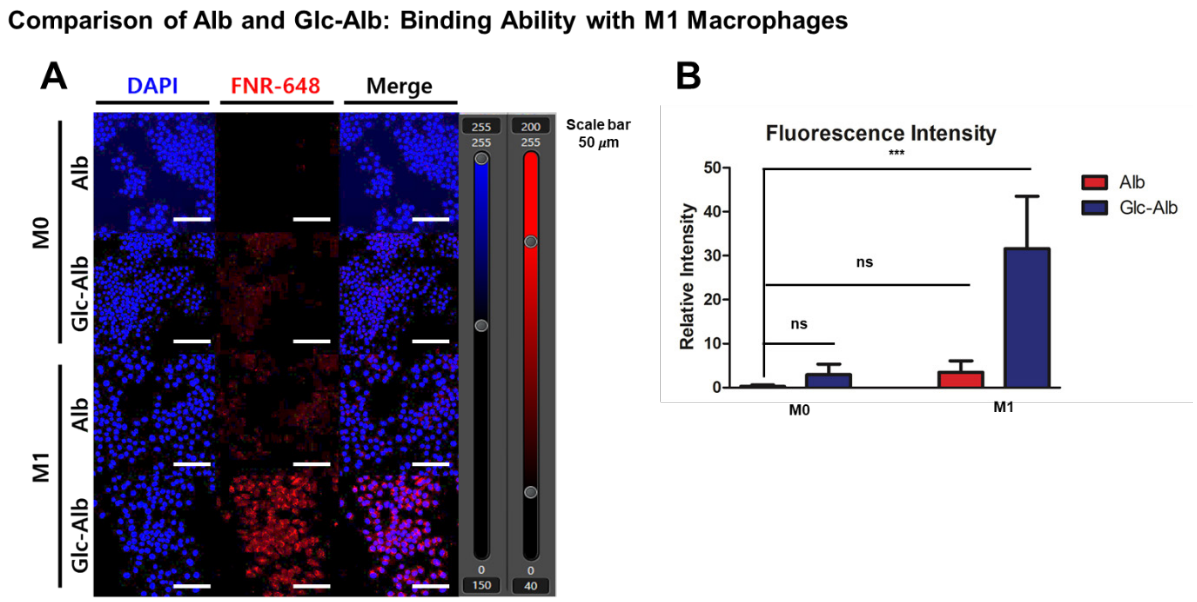

Figure S18. Comparison of Alb and Glc-Alb; Binding Ability with M1 Macrophages. (A) Red features shown in this image represent fluorescently-labeled albumin (B) Comparing to non-targetable albumin, GLUT1-targetable albumin conjugated with glucose demonstrated approximately 10 folds binding ability to M1 macrophages. Scale bar = 50 μm.

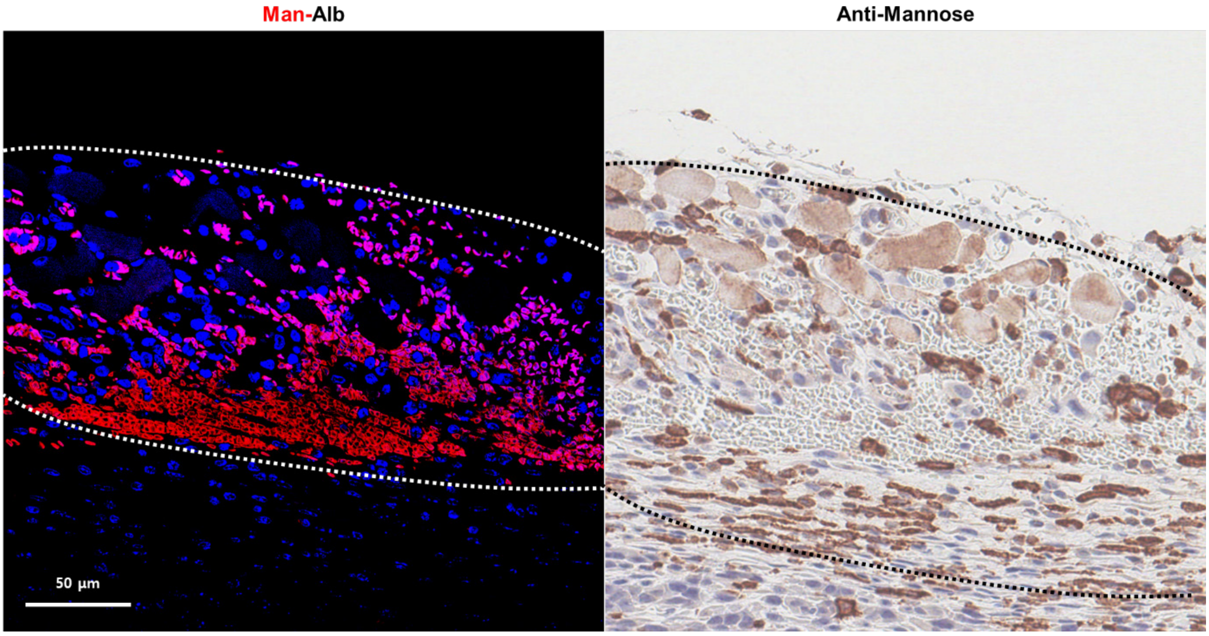

Figure S19. Micro-distribution of Man-Alb in tumor tissues. Representative microscopic fluorescence images of Man-Alb with Flamma Fluors 648 after 24 h intravenous administration in 4T1 tumor-bearing mouse-derived tumor tissue. Colocalization was assessed in comparison with adjacent sections stained with the anti-CD206 antibody. The white and black dotted line indicates the enlarged region in each image, and the area within the lines represents the TAM distribution.

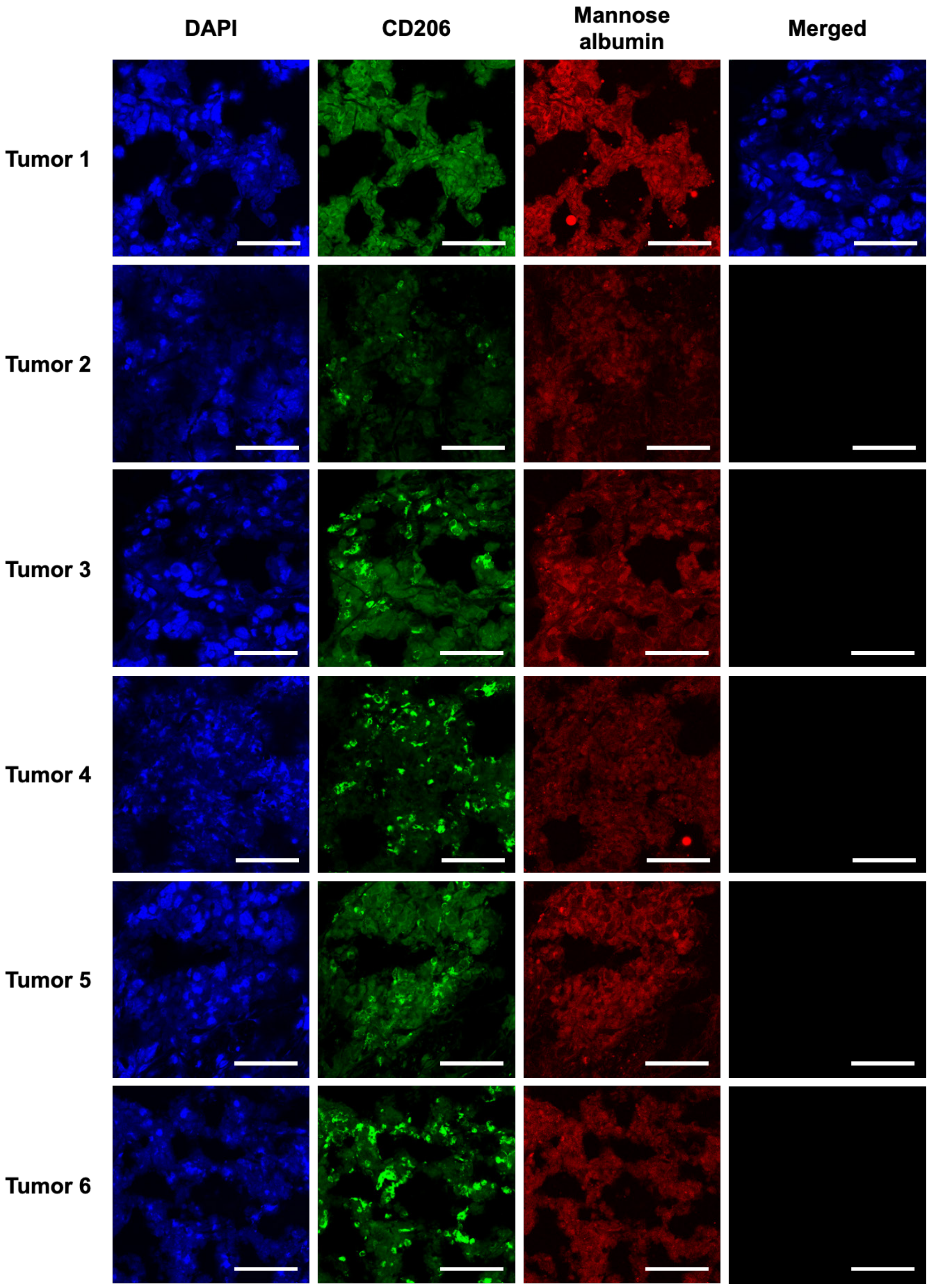

Figure S20. Six different mouse 4T1 tumors stained with anti-CD206 antibody excised after glycated albumin i.v. injection. To validate that mannosylated albumin targets the mannose receptor, we conducted verification at the protein level. Using immunofluorescence, we stained mannose receptors in 4T1 tumor tissues of mice injected with fluorescently conjugated Man-Alb and performed fluorescent imaging. As

a result, we observed a high Man-Alb uptake around the stained mannose receptors in six different tumors. Scale bar = 50 μm.

2. Supplemental Tables

Table S1. Average molecular weights measured using MALDI-TOF for control, represented by albumin, and samples obtained after individual reactions of 50 mg/0.5 mL albumin and ADIBO compound at a 1:11 ratio. Each sample underwent a separate reaction, and the results presented in the table depict the outcomes of each reaction. The table includes four samples: Sample 1, utilized as the control in the albumin experimental group according to the paper; Sample 2, introduced with mannose; Sample 3, introduced with galactose; Sample 4, introduced with glucose.

MALDI-TOF-Based DOF

Sample	Centroid Mass	Lower Bound	Upper Bound	Charge (z)	Height	Relative Intensity	Area
**Control**	66440.25	66422.21	66682.1	0	5613	100	545904.5
**Sample1**	69179.56	69156.23	69208.76	0	2025	92.51	32343.79
**Sample2**	69163.22	69141.91	69220.7	0	6244	98.72	46410.45
**Sample3**	69284.32	69266.08	69311.48	0	8561	100	70890.65
**Sample4**	69118.59	69083.31	69154.69	0	7968	98.61	23610.39

Sample	Centroid Mass	ADIBO DOF (MALDI-TOF)
**Control**	66440.25	Sample-control/Mw of ADIBO
**Sample1**	69179.56	7.62 (Albumin experimental group)
**Sample2**	69163.22	**7.58**
**Sample3**	69284.32	**7.91**
**Sample4**	69118.59	**7.45**

Table S2. Calculation of the degree of modification (DOF) for ADIBO-modified Albumin based on UV absorbance. In this process, the absorbance changes of albumin at A280 concerning the ADIBO concentration are converted to A280c using a correlation factor. The relevant formula utilized in this calculation is derived from the results of the previous paper[1,2].

UV-Based DOF

Sample	A280	A312	A280c	ADIBO DOF(UV)
**Control**	0.131		0.131	
**Sample1**	0.459	0.329	0.174	**6.14**
**Sample2**	0.442	0.321	0.163	**6.30**
**Sample3**	0.436	0.314	0.164	**6.15**
**Sample4**	0.410	0.295	0.154	**6.18**

Table S3. Molecular weights of the Mannose, Galactose, and Glucose-introduced samples (Samples 2, 3, and 4 respectively) were determined using MALDI-TOF. For each reaction sample, the measured molecular weight was subtracted from the previously determined molecular weight, and the result was divided by the molecular weight of the introduced sugar. This calculation was employed to compute the degree of modification (DOF) for each sample.

Rxn ratio	Sample	Centroid Mass	Carbohydrate DOF (MALDI-TOF)
**1:5**	**DOF=(Carbohydrate5-sample#)/Mw of Carbohydrate**		
	Man5 (using Sample2)	70609.8	**4.219540598**
	Gal5 (using Sample3)	70466.07	**3.793459313**
	Glc5 (using Sample4)	70077.17	**4.341510114**

Table S4. Quantitative values of the time-activity curve based on PET imaging.

Blood									
**Time (h)**	**Albumin**	**SD**	**Glc-Alb**	**SD**	**SD**	**Gal-Alb**	**SD**	**Man-Alb**	**SD**
**0**	43.27	2.35	42.59	1.86	1.06	42.98	1.22	45.97	0.80
**2**	37.00	1.71	38.00	2.20	1.05	24.34	1.77	18.80	1.17
**4**	30.55	1.51	32.88	2.43	1.66	18.84	1.13	10.75	2.19
**24**	9.99	1.86	7.34	1.39	1.60	5.74	1.10	5.33	0.48
**48**	4.39	1.17	2.31	0.50	0.35	2.47	0.57	2.07	0.15

Liver									
**Time (h)**	**Albumin**	**SD**	**Glc-Alb**	**SD**	**SD**	**Gal-Alb**	**SD**	**Man-Alb**	**SD**
**0**	10.62	2.00	9.06	0.71	0.64	15.70	1.15	9.67	0.77
**2**	15.59	0.68	11.12	0.60	1.84	32.68	2.48	32.43	1.74
**4**	20.59	1.19	15.74	1.06	0.98	36.17	2.03	36.07	1.42
**24**	13.11	2.47	7.59	1.25	0.94	6.54	2.20	19.05	2.05
**48**	10.35	1.51	3.80	0.95	0.22	2.33	0.57	14.43	0.90

Regions of interest (ROI) were defined for Blood and Liver, and %ID/g was calculated using nuclear medicine quantitative methods for images obtained immediately after injection (0 h), 2, 4, 24, and 48 h post-injection.

Table S5. In the *ex vivo* results, immediately after acquiring the 24 h PET images.

Blood and all organs were dissected, and the %ID/g for each organ was measured using a gamma counter.

	Man-Alb	SD	Alb	SD	Gal-Alb	SD	Glc-Alb	SD
**Blood**	3.45	0.17	7.77	1.01	4.07	0.43	3.81	0.33
**Muscle**	0.79	0.05	1.30	0.25	0.65	0.08	0.85	0.04
**Heart**	1.88	0.38	1.92	0.34	1.49	0.08	1.31	0.04
**Lung**	2.64	0.52	2.24	0.91	2.98	0.74	2.84	0.85
**Liver**	15.13	0.35	9.15	0.38	6.41	0.24	5.12	0.19
**Spleen**	2.66	0.48	2.11	0.35	2.18	0.11	2.21	0.20
**Stomach**	1.23	0.52	1.03	0.20	0.75	0.26	0.83	0.24
**Intestine**	1.63	0.08	1.88	0.50	1.64	0.15	1.75	0.09
**Kidney**	1.62	0.14	2.03	0.48	1.54	0.28	1.46	0.16

Table S6. Quantitative data obtained using *ex vivo* fluorescence imaging.

	Alb	SD
**T/Li**	1.349	0.02989
**T/Ki**	2.078183	0.110935
**T/He**	3.65346	0.195985
**T/Sp**	5.110407	0.284733
**T/In**	1.249868	0.122029
**T/Lu**	1.371896	0.066561

	Man-Alb	SD
**T/Li**	0.565	0.02148
**T/Ki**	1.759434	0.052017
**T/He**	2.495283	0.042453
**T/Sp**	3.693396	0.570755
**T/In**	0.740566	0.103774
**T/Lu**	2.391509	0.193396

	Gal-Alb	SD
**T/Li**	1.99113	0.058837
**T/Ki**	2.12803	0.106955
**T/He**	4.086407	0.287671
**T/Sp**	4.964173	1.032666
**T/In**	1.486301	0.055321
**T/Lu**	3.311907	0.733931

Table S7. Top 20 DEGs of cluster 0 acquired by UMAP clustering of the integrated dataset.

Gene	Average log fold change	Adjusted P-value
Pabpc1	462.518393	0
Hist1h3b	370.18591	0
Eef1b2	344.217399	0
Anxa2	339.889314	0
Hist1h3c	334.118526	0
Col1a1	332.675839	0
Rack1	315.363498	0
Txn1	313.920803	0
Anp32b	305.264633	0
Lgmn	296.589054	7.7863E-274
Ctsb	272.082136	1.6535E-292
Mtch1	260.089146	0
Lgals3	240.343534	2.7482E-129
Pkm	237.214277	2.2958E-158
Clic1	236.014787	0
Ncl	228.801796	0
Hist2h2bb	224.470139	0
Bsg	223.031016	2.534E-279
S100a4	221.588321	1.7605E-256
Atp5e	215.817606	0

Table S8. Top 20 DEGs of cluster 1 acquired by UMAP clustering of the integrated dataset.

Gene	Average log fold change	Adjusted P-value
Fn1	193.420832	0
Cd74	184.764672	0
Atp1a1	132.827153	0
Spp1	103.999868	0
H2-Ab1	96.729091	0
Gadd45a	69.8005463	0
Crlf1	45.8135811	0
Fetub	39.3854596	0
C1qc	38.9808894	0
Slco2a1	38.0745893	0
Plcg2	27.9497433	0
Cp	25.7466507	0
Coro6	25.4142485	0
Msmp	19.4160685	0
Tcaf1	18.9300661	0
Serpine2	18.8635833	0
Mgp	17.421734	0
Tle2	16.9833609	0
Npnt	15.7916612	0
Dock6	15.2131755	0

Table S9. Top 20 DEGs of cluster 2 acquired by UMAP clustering of the integrated dataset.

Gene	Average log fold change	Adjusted P-value
Col1a2	67.0553697	0
Mmp12	43.8256635	5.16E-298
Adm	56.9520813	1.26E-277
Ndrg1	34.9758824	1.66E-227
Mt1	232.965308	2.43E-199
Mmp141	2.13379539	7.89E-174
Nos2	5.38124927	2.10E-173
Rhov	1.64306943	3.15E-140
Tuba1a	6.21312894	3.96E-138
Hspa1b	82.7698205	7.45E-135
Hmox1	121.694647	2.12E-134
Bnip3	3.59558436	9.19E-127
Ero1l	12.3185678	7.11E-114
Eno2	7.3292148	1.96E-111
Espn	31.8147214	2.54E-96
Ackr3	4.87164018	1.32E-94
Smox	11.2038603	4.18E-93
Mt2	346.928519	5.91E-92
Myl12a	11.293452	1.75E-90
Ncs1	4.8897391	4.25E-86

Table S10. Top 20 DEGs of cluster 3 acquired by UMAP clustering of the integrated dataset.

Gene	Average log fold change	Adjusted P-value
Mybpc2	848.485441	0
Mylpf	750.829685	0
Ckm	721.458647	0
Myh1	621.992016	0
Atp2a1	418.571532	0
Tnnc2	386.831386	0
Myl1	271.233876	0
Ttn	254.104763	0
Actn3	235.338695	0
Pvalb	202.26938	0
Tpm2	194.9538	0
Gpx3	171.869806	0
Ankrd23	158.703789	0
Casq1	125.509903	0
Car3	92.9657898	0
Ryr1	90.5091599	0
Myom2	85.2908626	0
Cox6a2	82.6066988	0
Pdlim3	82.3972788	0
Cryab	80.9149009	0

Table S11. Top 20 DEGs of cluster 4 acquired by UMAP clustering of the integrated dataset.

Gene	Average log fold change	Adjusted P-value
Sfrp2	64.114409	0
Ptx3	16.3019456	0
Reg3g	42.1499559	7.05E-248
Prelp1	5.62774049	5.33E-185
Hp	11.0554994	8.34E-160
Cxcl12	14.7206391	9.33E-157
Map3k20	3.57653057	4.19E-153
Sfrp1	36.5818315	2.03E-135
Lbp	9.26968533	1.12E-123
Upf11	0.59364759	1.26E-114
Stt3b1	0.28010334	8.80E-112
Zdhhc32	0.36694364	3.57E-100
Cyp1b1	18.94735	3.77E-100
Clint11	0.5564319	4.42E-100
Isyna11	1.52588692	1.16E-96
Ifitm2	4.14559755	2.53E-95
Ccn51	4.7179963	5.87E-92
Hspa131	0.85650185	1.66E-88
Efemp1	1.29916868	1.27E-84
Mettl261	0.77546411	2.46E-83

Table S12. Correlation analysis between each cell type acquired by CellDART analysis and fluorescence intensities in all spots of the Man+Gal sample. (A) Correlation coefficients and (B) p-values.

(A) Correlation coefficients

Cell type	red (mannose)	green (galactose)
Inflammatory macrophage	0.2208696	0.33223033
Endothelial	-0.1200567	-0.0961463
Neutrophil	0.13491602	0.2482879
Proliferative myeloid	-0.2341376	-0.2847813
Monocyte-derived cell	0.07282752	0.0094533
Fibroblast	-0.0729248	-0.061613
Epithelial/Cancer cell	-0.1045646	-0.0250167
T cell/NK cell	0.00958025	-0.0386518
Anti-inflammatory macrophage	0.12601342	-0.0184209
Tumor-associated macrophage	0.13720882	0.00730360

(B) p-values

Cell type	red (mannose)	green (galactose)
Inflammatory macrophage	4.23112801007331E-37	3.03145769543748E-88
Endothelial	6.25429577842064E-10	6.49897355637674E-06
Neutrophil	7.10726100151483E-13	1.66063252704574E-47
Proliferative myeloid	5.73999461988404E-42	1.62712001756707E-63
Monocyte-derived cell	0.00740752174436353	1
Fibroblast	0.00723083474510885	0.107877542756837
Epithelial/Cancer cell	3.15315353572115E-07	1

T cell/NK cell	1	1
Anti-inflammatory macrophage	4.55263462044097E-11	1
Tumor-associated macrophage	3.16034869618611E-16	0.665248196824856

Table S13. Correlation analysis between each cell type acquired by CellDART analysis and fluorescence intensities in cluster 0 of the Man+Gal sample. (A) Correlation coefficients and (B) p-values.

(A) Correlation coefficients

Cell type	red (mannose)	green (galactose)
Inflammatory macrophage	0.03811764	-0.0237734
Endothelial	-0.0875341	-0.0360649
Neutrophil	0.01303532	-0.0231246
Proliferative myeloid	0.04546823	0.05230927
Monocyte-derived cell	-0.0833108	0.02320275
Fibroblast	-0.1248545	-0.0345513
Epithelial/Cancer cell	0.08528971	-0.0231502
T cell/NK cell	0.02269597	-0.0131371
Anti-inflammatory macrophage	-0.0316974	0.02823828
Tumor-associated macrophage	-0.01000232	0.05047250

(B) P-values

Cell type	red (mannose)	green (galactose)
Inflammatory macrophage	1	1
Endothelial	0.260210440898932	1
Neutrophil	1	1
Proliferative myeloid	1	1
Monocyte-derived cell	0.3367136099181	1
Fibroblast	0.00534864221128528	1
Epithelial/Cancer cell	0.30068418263306	1

T cell/NK cell	1	1
Anti-inflammatory macrophage	1	1
Tumor-associated macrophage	0.76015031750125	0.123212693634834

Table S14. Correlation analysis between each cell type acquired by CellDART analysis and fluorescence intensities in cluster 1 of the Man+Gal sample. (A) Correlation coefficients and (B) p-values.

(A) Correlation coefficients

Cell type	red (mannose)	green (galactose)
Inflammatory macrophage	0.09248446	0.10613744
Endothelial	-0.0445376	-0.0231407
Neutrophil	0.02952914	0.04581126
Proliferative myeloid	-0.113838	-0.0834962
Monocyte-derived cell	0.05972895	0.06083096
Fibroblast	-0.0135183	-0.0121247
Epithelial/Cancer cell	-0.16926	-0.1522331
T cell/NK cell	0.09435649	0.03350749
Anti-inflammatory macrophage	0.12111101	0.0833515
Tumor-associated macrophage	0.13525457	0.06668253

(B) p-values

Cell type	red (mannose)	green (galactose)
Inflammatory macrophage	0.0240238568731797	0.00413272494384935
Endothelial	1	1
Neutrophil	1	1
Proliferative myeloid	0.00131947151044537	0.0656966154548174
Monocyte-derived cell	0.504773238052031	0.485122401991637
Fibroblast	1	1
Epithelial/Cancer cell	3.56313013600334E-08	1.42163041605343E-06

T cell/NK cell	0.0194601216707521	1
Anti-inflammatory macrophage	0.000418795243746945	0.0656966154548174
Tumor-associated macrophage	1.02416243552139E-06	0.0163535790620539

Table S15. Correlation analysis between each cell type acquired by CellDART analysis and fluorescence intensities in cluster 2 of the Man+Gal sample. (A) Correlation coefficients and (B) p-values.

(A) Correlation coefficients

Cell type	red (mannose)	green (galactose)
Inflammatory macrophage	0.14320125	0.26770146
Endothelial	-0.0979828	-0.1093521
Neutrophil	0.04222735	0.15457345
Proliferative myeloid	-0.018573	-0.090942
Monocyte-derived cell	0.05492572	-0.158157
Fibroblast	-0.0381338	-0.0476959
Epithelial/Cancer cell	-0.1260745	0.00302298
T cell/NK cell	-0.0862531	-0.0383306
Anti-inflammatory macrophage	-0.056322	-0.2201301
Tumor-associated macrophage	-0.01259236	-0.18221828

(B) p-values

Cell type	red (mannose)	green (galactose)
Inflammatory macrophage	0.00852008185353649	8.7259379058087E-11
Endothelial	0.369334696916565	0.160223602034953
Neutrophil	1	0.00259096394574099
Proliferative myeloid	1	0.59779186919471
Monocyte-derived cell	1	0.00176085252562932
Fibroblast	1	1
Epithelial/Cancer cell	0.0432070714509456	1

T cell/NK cell	0.800946369583814	1
Anti-inflammatory macrophage	1	4.11861081038958E-07
Tumor-associated macrophage	0.743996314007519	1.88529014960909E-06

Table S16. Correlation analysis between each cell type acquired by CellDART analysis and fluorescence intensities in cluster 3 of the Man+Gal sample. (A) Correlation coefficients and (B) p-values.

(A) Correlation coefficients

Cell type	red (mannose)	green (galactose)
Inflammatory macrophage	-0.1006805	-0.1699513
Endothelial	-0.0494646	0.05095446
Neutrophil	-0.0549648	0.07083611
Proliferative myeloid	-0.1470101	-0.2624883
Monocyte-derived cell	0.12202184	0.23150563
Fibroblast	-0.0766019	0.11496541
Epithelial/Cancer cell	-0.1747156	-0.2692203
T cell/NK cell	7.42E-05	-0.0159101
Anti-inflammatory macrophage	0.28265431	0.13412131
Tumor-associated macrophage	0.30254787	0.21808190

(B) p-values

Cell type	red (mannose)	green (galactose)
Inflammatory macrophage	0.697854191234756	0.00814101290306153
Endothelial	1	1
Neutrophil	1	1
Proliferative myeloid	0.0431980679899234	3.64878204727616E-07
Monocyte-derived cell	0.226561287462254	1.74686553011355E-05
Fibroblast	1	0.328386483515848

Epithelial/Cancer cell	0.00548629890982998	1.47657622936119E-07
T cell/NK cell	1	1
Anti-inflammatory macrophage	2.24367360231776E-08	0.103775946085279
Tumor-associated macrophage	2.09485331160246E-11	1.81609471514083E-06

Table S17. Correlation analysis between each cell type acquired by CellDART analysis and fluorescence intensities in cluster 4 of the Man+Gal sample. (A) Correlation coefficients and (B) p-values.

(A) Correlation coefficients

Cell type	red (mannose)	green (galactose)
Inflammatory macrophage	-0.0422479	0.00398204
Endothelial	-0.1056996	-0.0044945
Neutrophil	0.02501233	0.15817267
Proliferative myeloid	-0.199417	-0.1084002
Monocyte-derived cell	0.05907944	0.02196695
Fibroblast	-0.0271091	0.06546152
Epithelial/Cancer cell	-0.1247569	0.02195726
T cell/NK cell	-0.0158513	-0.1269124
Anti-inflammatory macrophage	0.21326355	-0.0193096
Tumor-associated macrophage	0.11466441	-0.05259800

(B) p-values

Cell type	red (mannose)	green (galactose)
Inflammatory macrophage	1	1
Endothelial	1	1
Neutrophil	1	1
Proliferative myeloid	0.837573855894653	1
Monocyte-derived cell	1	1
Fibroblast	1	1
Epithelial/Cancer cell	1	1

T cell/NK cell	1	1
Anti-inflammatory macrophage	0.555456880479747	1
Tumor-associated macrophage	0.182131636189465	0.541579894075019

Table S18. Top 20 DEGs of mannose albumin distinct region in the Man+Gal sample acquired by IAMSAM analysis.

Gene	Average log fold change	Adjusted P-value
Gzmg	-27.708012	4.64E-07
Fgf17	-26.411606	3.23E-06
Hist1h2aa	-26.086918	0.00018604
Lrrc75b	-25.468466	0.0079021
1700007K13Rik	-25.454277	0.00503621
Tnk1	-25.383005	0.00364662
Axdnd1	-25.016735	0.00918572
Egr3	-24.86996	0.03169945
Rpgrip1l	-24.833708	0.03657398
Tfap2c	-24.677794	0.04604627
Psg19	-24.493507	0.04885401
1700061G19Rik	-5.9501824	2.31E-05
Ptx3	5.5664077	4.22E-38
Sfrp2	5.3996825	4.60E-72
Arg1	-5.3827386	6.37E-40
Acta1	5.2867875	7.59E-65
Nudt17	-5.107946	0.00457024
Myh4	5.0715494	4.78E-57
Bdh2	-4.96761	0.00014907
Cyp24a1	-4.961956	1.07E-09

Table S19. Top 20 DEGs of galactose albumin distinct region in the Man+Gal sample acquired by IAMSAM analysis.

Gene	Average log fold change	Adjusted P-value
Sypl2	-27.556383	1.12E-05
Eef1a2	-27.527044	1.49E-07
Ldb3	-27.393818	3.75E-05
Fitm1	-27.188604	4.09E-05
Myom1	-26.955032	3.15E-05
Ppp1r3c	-26.907875	0.00110651
Myh8	-26.813883	0.00024069
Alpk3	-26.72168	9.33E-06
Igfn1	-26.71516	0.00979161
Ckmt2	-26.641422	9.15E-05
Kcnc4	-26.553402	0.00069335
3425401B19Rik	-26.358194	0.0001125
Ampd1	-26.348711	0.01007747
Nol3	-26.33112	0.00083155
Card14	-26.282255	0.00020563
Prkaa2	-26.222946	0.00167692
Abra	-26.045801	0.00251146
Wfdc1	-25.979502	0.01128804
Musk	-25.961615	0.00421213
Entpd2	-25.884182	1.94E-05

Table S20. Table of log fold change and adjusted p-values for comparing gene expression levels of each glycan binding gene between segmented ROI from IAMSAM and the rest region in the Man+Gal sample (Man-Alb).

Gene	Log fold change	Adjusted P-value
Cd22	0.60316180	5.404920e-01
Cd302	0.44849410	1.531252e-01
Cd33	-0.86683810	3.160000e-11
Clec10a	1.22249250	4.454560e-01
Clec11a	2.66377450	2.218784e-03
Clec12a	0.66013070	4.971729e-01
Clec14a	1.66566200	5.540189e-01
Clec2d	0.88393617	6.050000e-06
Clec2f	-1.67409700	1.460000e-12
Clec3b	2.81141730	3.370000e-10
Clec5a	-1.40326750	1.860000e-12
Clec9a	-1.06391070	3.722578e-01
Lgals1	-0.68183017	2.420000e-14
Lgals3	-1.79610060	4.260000e-45
Lgals7	-5.76072600	4.410000e-19
Lgals8	-0.69633126	2.950000e-07
Lgals9	-1.45718150	8.750000e-18
Mrc2	1.67144900	7.470000e-14
Sell	0.53212870	4.519476e-01

Selp	2.17513300	8.263895e-03
Siglec1	0.01836025	5.042569e-01

Table S21. Log fold change and adjusted p-values for comparing gene expression levels of each glycan binding gene between segmented ROI from IAMSAM and the rest region in the Man+Gal sample (Gal-Alb).

Gene	Log fold change	Adjusted P-value
Cd22	-2.3292944	7.716649e-02
**Cd302**	-1.9593334	1.340000e-09
**Cd33**	-0.1591553	1.780000e-11
**Clec10a**	-1.4377073	6.800000e-09
Clec11a	-0.4300078	5.300422e-02
**Clec12a**	-2.0071967	2.096190e-04
**Clec14a**	-3.4634254	6.370000e-08
**Clec2d**	-1.1688634	4.490000e-40
**Clec2f**	-0.7378714	3.980000e-18
**Clec3b**	-3.2967920	4.930000e-05
**Clec5a**	-0.9854431	1.010000e-20
Clec9a	-2.8067646	5.711285e-02
**Lgals1**	-0.9389442	8.320000e-42
**Lgals3**	0.5725325	2.360000e-34
**Lgals7**	0.4316750	4.590618e-03
**Lgals8**	-0.8590883	1.100000e-29
**Lgals9**	-0.9528802	3.150000e-31
**Mrc2**	-1.8751295	8.230000e-48

Sell	0.3201100	9.378173e-02
**Selp**	-2.3125925	9.210000e-07
Siglec1	-0.8177713	6.713155e-02

Table S22. Quantitative values of the time-activity curve based on PET imaging. Regions of interest (ROI) were defined for blood and liver, and %ID/g was calculated using nuclear medicine quantitative methods for images obtained immediately after injection (0 h), 4, 8, and 24 h post-injection.

Blood								
**Time (h)**	**Albumin**	**SD**	**Glc(2)-Alb**	**SD**	**Glc(6)-Alb**	**SD**	**Man-Alb**	**SD**
**0**	43.09	1.53	43.46	0.86	53.27	0.79	44.11	3.29
**4**	31.32	2.65	29.17	0.86	43.64	0.84	12.24	0.99
**8**	19.85	1.22	21.47	0.97	33.47	1.10	7.01	0.46
**24**	4.75	0.71	7.18	0.76	10.57	2.49	3.02	0.13

Liver								
**Time (h)**	**Albumin**	**SD**	**Glc(2)-Alb**	**SD**	**Glc(6)-Alb**	**SD**	**Man-Alb**	**SD**
**0**	14.23	1.51	9.20	0.94	12.47	1.70	15.17	1.21
**4**	22.31	1.25	15.31	0.79	21.61	1.39	33.68	1.39
**8**	25.13	1.82	16.56	0.56	29.10	1.38	39.44	1.27
**24**	6.47	0.63	5.42	0.66	12.36	0.61	21.13	1.99

Tumor								
**Time (h)**	**Albumin**	**SD**	**Glc(2)-Alb**	**SD**	**Glc(6)-Alb**	**SD**	**Man-Alb**	**SD**
**0**	0.80	0.38	0.32	0.18	0.98	0.16	0.30	0.10
**4**	1.14	0.37	0.47	0.10	1.55	0.21	1.69	0.16
**8**	1.60	0.40	0.83	0.15	2.68	0.33	2.24	0.23
**24**	2.64	0.15	1.99	0.22	4.80	0.58	2.46	0.37

Table S23. In the *ex vivo* results, immediately after acquiring the 24 h PET images. Blood and all organs were dissected, and the %ID/g for each organ was measured using a gamma counter.

	Alb	SD	G2-Alb	SD	G6-Alb	SD	Man-Alb	SD
Blood	3.31	0.41	2.89	0.31	3.07	0.28	3.21	0.28
Heart	0.26	0.01	0.31	0.03	0.45	0.02	0.2	0.01
Lung	0.53	0.12	0.63	0.03	0.73	0.21	0.41	0.19
Liver	5.87	0.7	3.68	0.71	6.41	0.48	12.58	0.51
Spleen	1.93	0.07	1.92	0.38	1.61	0.4	1.88	0.36
Stomach	0.16	0.01	0.4	0.04	0.52	0.04	0.14	0.06
Intestine	2.79	0.2	3.52	0.63	6.45	0.8	1.47	0.12
Kidney	1.18	0.06	1.12	0.05	1.05	0.21	1.2	0.11
Tumor	2.51	0.8	2	0.23	4.84	0.67	2.38	0.3

Table S24. Correlation analysis between each cell type acquired by CellDART analysis and fluorescence intensities in all spots of the Man+Glc sample. (A) Correlation coefficients and (B) p-values.

(A) Correlation coefficients

Cell type	red (mannose)	green (glucose)
Inflammatory macrophage	-0.0698063	0.07813957
Endothelial	0.00688675	-0.0481746
Neutrophil	0.0653031	0.25472497
Proliferative myeloid	-0.2430933	-0.2288346
Monocyte-derived cell	0.02920878	-0.0060041
Fibroblast	0.17779465	0.0029509
Epithelial/Cancer cell	-0.1629624	0.03560266
T cell/NK cell	-0.0409379	0.01156201
Anti-inflammatory macrophage	0.3964213	0.13374418
Tumor-associated macrophage	0.43874871	0.16593661

(B) p-values

Cell type	red (mannose)	green (glucose)
Inflammatory macrophage	0.0412417599668372	0.00576342774206779
Endothelial	1	1
Neutrophil	0.10760772043008	1.4821738449444E-44
Proliferative myeloid	2.36428411499048E-40	1.68362722704621E-35
Monocyte-derived cell	1	1
Fibroblast	9.91091548609438E-21	1
Epithelial/Cancer cell	3.50162566439376E-17	1

T cell/NK cell	1	1
Anti-inflammatory macrophage	3.30139914678584E-115	3.96990971363928E-11
Tumor-associated macrophage	2.37154623471946E-147	9.47883208660712E-21

Table S25. Correlation analysis between each cell type acquired by CellDART analysis and fluorescence intensities in cluster 0 of the Man+Glc sample. (A) Correlation coefficients and (B) p-values.

(A) Correlation coefficients

Cell type	red (mannose)	green (glucose)
Inflammatory macrophage	-0.1727889	0.12634797
Endothelial	0.18523196	-0.0263493
Neutrophil	0.00735779	0.12030554
Proliferative myeloid	-0.0650674	0.08536607
Monocyte-derived cell	-0.1673274	-0.0297645
Fibroblast	0.28371299	-0.0962097
Epithelial/Cancer cell	-0.0198643	-4.59E-05
T cell/NK cell	-0.1067481	0.02693247
Anti-inflammatory macrophage	-0.0601388	-0.0771791
Tumor-associated macrophage	-0.03023438	-0.15354023

(B) p-values

Cell type	red (mannose)	green (glucose)
Inflammatory macrophage	5.77585510277272E-08	0.000386346959202606
Endothelial	3.46591907850525E-09	1
Neutrophil	1	0.000942644048316394
Proliferative myeloid	0.389757712438182	0.073357480189143
Monocyte-derived cell	1.79306364954041E-07	1
Fibroblast	2.62160530120889E-22	0.0212321518126556
Epithelial/Cancer cell	1	1

T cell/NK cell	0.00595781622637377	1
Anti-inflammatory macrophage	0.533257373994597	0.145951939349586
Tumor-associated macrophage	0.290937837491366	6.8966136935486E-08

Table S26. Correlation analysis between each cell type acquired by CellDART analysis and fluorescence intensities in cluster 1 of the Man+Glc sample. (A) Correlation coefficients and (B) p-values.

(A) Correlation coefficients

Cell type	red (mannose)	green (glucose)
Inflammatory macrophage	-0.0269478	0.08810371
Endothelial	0.12173464	-0.0503923
Neutrophil	0.15067978	0.07497714
Proliferative myeloid	-0.1497584	-0.0297661
Monocyte-derived cell	-0.2485259	-0.0328042
Fibroblast	0.55340483	-0.0619
Epithelial/Cancer cell	0.00794727	0.06451901
T cell/NK cell	-0.051284	0.05447116
Anti-inflammatory macrophage	-0.0490228	-0.0038295
Tumor-associated macrophage	-0.15956957	-0.03653125

(B) p-values

Cell type	red (mannose)	green (glucose)
Inflammatory macrophage	1	0.393846273648761
Endothelial	0.0189227803707314	1
Neutrophil	0.000621953216627413	0.870479849816727
Proliferative myeloid	0.000687670066512024	1
Monocyte-derived cell	2.45440474306284E-11	1
Fibroblast	1.01560336787153E-65	1

Epithelial/Cancer cell	1	1
T cell/NK cell	1	1
Anti-inflammatory macrophage	1	1
Tumor-associated macrophage	3.99817347620103E-06	0.294034308368256

Table S27. Correlation analysis between each cell type acquired by CellDART analysis and fluorescence intensities in cluster 2 of the Man+Glc sample. (A) Correlation coefficients and (B) p-values.

(A) Correlation coefficients

Cell type	red (mannose)	green (glucose)
Inflammatory macrophage	0.08435958	-0.0438793
Endothelial	0.1503489	0.03540155
Neutrophil	0.12998638	0.15315145
Proliferative myeloid	-0.1224943	-0.1225398
Monocyte-derived cell	-0.2333041	-0.2663588
Fibroblast	0.34799788	0.24234794
Epithelial/Cancer cell	0.0038493	0.16531502
T cell/NK cell	0.15562365	0.17705297
Anti-inflammatory macrophage	-0.0290969	-0.0779194
Tumor-associated macrophage	0.05387025	-0.03498392

(B) p-values

Cell type	red (mannose)	green (glucose)
Inflammatory macrophage	1	1
Endothelial	0.116279438993331	1
Neutrophil	0.35576658026363	0.0995587123787049
Proliferative myeloid	0.504770541412502	0.504770541412502
Monocyte-derived cell	0.000191109742920989	6.65330385915228E-06
Fibroblast	1.35376873732519E-10	8.18668732442138E-05
Epithelial/Cancer cell	1	0.048242695326672

T cell/NK cell	0.0868127213591736	0.0221442826683556
Anti-inflammatory macrophage	1	1
Tumor-associated macrophage	0.29235916932123	0.494280569349126

Table S28. Correlation analysis between each cell type acquired by CellDART analysis and fluorescence intensities in cluster 3 of the Man+Glc sample. (A) Correlation coefficients and (B) p-values.

(A) Correlation coefficients

Cell type	red (mannose)	green (glucose)
Inflammatory macrophage	0.02933961	0.06236991
Endothelial	-0.0577128	-0.0252773
Neutrophil	0.14769601	0.24869584
Proliferative myeloid	-0.1283576	-0.3229961
Monocyte-derived cell	0.11581205	0.24140858
Fibroblast	0.02922078	0.02062189
Epithelial/Cancer cell	-0.1522838	-0.2048598
T cell/NK cell	-0.0134078	-0.0157537
Anti-inflammatory macrophage	0.2239575	0.24498825
Tumor-associated macrophage	0.21891233	0.29274262

(B) p-values

Cell type	red (mannose)	green (glucose)
Inflammatory macrophage	1	1
Endothelial	1	1
Neutrophil	0.756149145090971	0.00465092518730416
Proliferative myeloid	1	1.51949264788324E-05
Monocyte-derived cell	1	0.00728398394367295
Fibroblast	1	1

Epithelial/Cancer cell	0.639814417489457	0.0594268783850984
T cell/NK cell	1	1
Anti-inflammatory macrophage	0.0207312498940157	0.0058571605853751
Tumor-associated macrophage	0.000573544316694848	3.29352852789186E-06

Table S29. Correlation analysis between each cell type acquired by CellDART analysis and fluorescence intensities in cluster 4 of the Man+Glc sample. (A) Correlation coefficients and (B) p-values.

(A) Correlation coefficients

Cell type	red (mannose)	green (glucose)
Inflammatory macrophage	-0.0619977	-0.0178203
Endothelial	-0.1136993	-0.0407802
Neutrophil	-0.039975	-0.0189341
Proliferative myeloid	-0.1115889	-0.1232751
Monocyte-derived cell	0.23228958	0.13987807
Fibroblast	-0.2878134	-0.1541618
Epithelial/Cancer cell	-0.243169	-0.1376001
T cell/NK cell	-0.0729575	-0.0560408
Anti-inflammatory macrophage	0.35091458	0.17803249
Tumor-associated macrophage	0.37849570	0.22662962

(B) p-values

Cell type	red (mannose)	green (glucose)
Inflammatory macrophage	1	1
Endothelial	0.361173662293343	1
Neutrophil	1	1
Proliferative myeloid	0.390684745213169	0.219372149213124
Monocyte-derived cell	2.80962267998009E-05	0.0844249119219931
Fibroblast	2.55901819716026E-08	0.0337180295716958
Epithelial/Cancer cell	8.37484571981802E-06	0.0955189124507059

T cell/NK cell	1	1
Anti-inflammatory macrophage	9.11344268190142E-13	0.00533764984910298
Tumor-associated macrophage	8.25389325970857E-17	1.15656285479403E-06

Table S30. Top 20 DEGs of mannose albumin distinct region in the Man+Glc sample acquired by IAMSAM analysis.

Gene	Average log fold change	Adjusted P-value
Ryr1	-27.08674	0.00015897
Obscn	-26.984142	6.30E-05
Myom2	-26.960209	1.17E-05
Neb	-26.88907	6.92E-05
Lmod2	-26.828676	9.16E-05
Trim63	-26.815748	2.90E-05
Mb	-26.724827	0.0036915
Actn2	-26.531425	0.00176931
Myf6	-26.451464	0.00356291
Csrp3	-26.293951	0.01539859
Art1	-26.22126	0.00645083
Trdn	-26.160881	0.00788211
Cmya5	-25.997904	0.02338126
Igfn1	-25.909643	0.03568442
Dusp13	-25.699266	0.04085212
Myot	-25.666935	0.02550295
Cyp24a1	-25.431581	0.00012084
Cldn2	-24.86121	0.00958655
Lypd5	-24.855042	0.00645083
Ido2	-24.696287	0.00928272

Table S31. Top 20 DEGs of glucose albumin distinct region in the Man+Glc sample acquired by IAMSAM analysis.

Gene	Average log fold change	Adjusted P-value
Prelp	-27.404818	0.0042486
Ankrd2	-26.795324	0.02648519
Hist1h2ba	-25.125332	0.03069904
Ptx3	-7.136006	0.00026893
Obscn	-6.523801	0.01706444
Lmod2	-6.32397	0.02007463
Trim63	-6.304115	0.01222639
Tcap	-6.228985	0.03005962
Reg3g	-6.1697164	0.0403946
Sfrp2	-6.1157546	2.13E-07
Mybpc2	-5.8416657	0.00344751
Pgam2	-5.6637855	0.04290238
Adamts5	-5.548464	0.00692082
1700007K13Rik	-5.4391217	0.005007
Serpina3n	-5.411527	8.87E-07
Lox	-5.3980246	7.77E-07
Nrap	-5.3871655	0.02295072
Col27a1	-5.339741	0.00576533
Slc22a17	-5.2932577	0.01021961
Car3	-5.209618	0.00399801

Table S32. Table of log fold change and adjusted p-values for comparing gene expression levels of each glycan binding gene between segmented ROI from IAMSAM and the rest region in the Man+Glc sample (Man-Alb).

Gene	Log fold change	Adjusted P-value
Cd302	0.425256220	1.283988e-01
**Cd33**	-0.095796420	1.170000e-05
Clec10a	1.415835700	7.375357e-01
Clec11a	1.247565300	9.823175e-02
**Clec12a**	-0.450702340	3.900000e-05
Clec14a	1.523555800	6.573463e-02
**Clec2d**	0.004300232	1.840000e-06
**Clec2f**	-0.366796500	9.940000e-07
Clec3b	0.685548100	8.051687e-01
**Clec5a**	-0.770137370	2.960000e-19
Clec9a	-0.215233240	2.786485e-01
**Lgals1**	-0.193946060	1.600000e-09
**Lgals3**	-1.014675100	3.700000e-77
**Lgals7**	-1.497322200	1.510000e-37
**Lgals8**	-0.393041800	2.350000e-17
**Lgals9**	-0.453236160	8.450000e-15
**Mrc2**	1.170958400	6.490000e-22
**Sell**	-0.487499620	8.460000e-08
Selp	1.400256300	3.119186e-01
Siglec1	0.639433740	7.085010e-01

Table S33. Table of log fold change and adjusted p-values for comparing gene expression levels of each glycan binding gene between segmented ROI from IAMSAM and the rest region in the Man+Glc sample (Glc-Alb).

Gene	Log fold change	Adjusted P-value
Cd302	-0.954647300	1.769481e-02
Cd33	-0.205099100	4.176238e-02
Clec10a	-1.842405900	2.660366e-02
Clec11a	-1.057535300	2.836440e-01
Clec12a	-0.244029670	2.589149e-01
Clec14a	-1.548401800	2.534055e-01
Clec2d	0.261604400	2.261141e-02
**Clec2f**	-0.286780180	3.223163e-03
Clec3b	-2.067606000	3.599218e-01
Clec5a	-0.306318730	7.050419e-02
Clec9a	-0.006824563	7.623977e-01
**Lgals1**	-0.590518100	1.280000e-23
**Lgals3**	1.026494400	1.720000e-37
**Lgals7**	-0.517907900	9.509929e-03
Lgals8	-0.225743170	1.099195e-01
Lgals9	-0.019747267	8.326610e-01
**Mrc2**	-1.988829700	8.710000e-21
Sell	0.831454040	3.747618e-01
Selp	-1.440586700	2.561801e-01
Siglec1	-2.111564900	1.511863e-01
